# Effects of Starch Synthesis-Related Genes Polymorphism on Quality of Glutinous Rice

**DOI:** 10.3389/fpls.2021.707992

**Published:** 2021-08-06

**Authors:** Ouling Zhang, Cheng Liang, Bowen Yang, Hui You, Liang Xu, Yongjun Chen, Xunchao Xiang

**Affiliations:** ^1^Lab of Plant Molecular Genetics and Breeding, Southwest University of Science and Technology, Mianyang, China; ^2^Rice Research Institute of Southwest University of Science and Technology, Mianyang, China

**Keywords:** glutinous rice (*Oryza sativa* L.), starch synthesis-related genes, targeted-gene association analysis, gene interaction, rice quality, *soluble starch synthase IIa*

## Abstract

Glutinous rice (*Oryza sativa* L.) quality includes thermal properties, retrogradation and pasting viscosity properties, and so on, which have little or no amylose. However, the genetic network regulation of different quality indices has not been systematically studied. The aim was to investigate the relationship between starch synthesis-related genes (SSRGs) and the physicochemical properties of glutinous rice by targeted-gene association analysis (TGAS). The genotypes of 17 SSRGs were analyzed using 46 gene-specific molecular markers in 63 glutinous rice accessions. TGAS and gene interactions analysis indicated that *soluble starch synthase* (*SS*) *IIa, SSI, starch branching enzyme* (*BE*) *IIa*, and *pullulanase* (*PUL*) had significant genetic effects on glutinous rice quality. *SSI* and *SSIIa* were the major genes that regulated thermal properties and retrogradation properties (RP). *PUL* was central in the regulation of gel consistency (GC), and it participated in the regulation of pasting viscosity parameters (PVP) except for the pasting time and the pasting temperature. *BEIIb, ISA1, SSIVb, BEIIa, SSIVa*, and their interactions with *SSIIa* regulated gelatinization temperature (GT) and PVP. The starch properties of glutinous rice are mainly controlled by *SSIIa, SSI, PUL*, and their interactions, but *SSIIa* is central among them. These findings indicate that starch properties in glutinous rice have a complex genetic system. It provides crucial information for promoting glutinous rice quality.

## Introduction

Rice (*Oryza sativa* L.) provides the staple food for nearly half the population of the world as one of the three major food crops in the world. Due to the successful utilization of the semi-dwarf gene and heterosis technology, rice yields have dramatically increased over the past several decades. Most research focused on the grain quality of non-glutinous rice. Rice starch is composed of two polysaccharides: amylose and amylopectin. Starch biosynthesis is a complex system involving 18 starch synthesis-related genes (SSRGs). It consists of four classes of enzymes: ADP-glucose pyrophosphorylase (AGP), starch synthase, branching enzyme (BE), and debranching enzyme (DBE) (Nakamura, 2002; James et al., 2003). However, glutinous rice (*Oryza sativa* L. var. *Glutinosa* Matsum) is a significant type of cultivated rice with long-standing cultural importance in Asia. Glutinous rice grains are milky white, have strong viscosity, and are slightly swollen in appearance. In glutinous rice, the *Waxy* (*Wx*) gene is mutated as the recessive *wx* gene, and no granule-bound starch synthase I (GBSSI) is produced. The production of the *wx* gene of glutinous rice is mainly due to a 23 bp insertion at the second exon of *Wx*, which leads to the termination of translation. Its mRNA cannot be translated into a biologically functional protein, which causes the loss of function of GBSSI, and ultimately, results in endosperm lacks or only with a little amylose content (AC) (≤ 2%) and contains essentially all amylopectin (Inukai et al., 2000; Musyoki et al., 2015). There is growing interest in understanding the genetic basis of glutinous rice quality and providing theoretical support for its quality improvement.

Amylopectin is synthesized by multiple isoforms of the four classes of enzymes: AGP, *soluble starch synthase* (SS), BE, and DBE. The structure of amylopectin from cultivated rice can be classified into the L-type (*indica*) and the S-type (*japonica*) because of the proportional difference of short chains of DP ≤10, in which the former was specifically lower than the latter (Nakamura, 2002). It is generally considered that eating and cooking qualities (ECQs) are the most crucial qualities of rice, which are mainly determined by the apparent amylose content (AAC), gel consistency (GC), and gelatinization temperature (GT) (Yan et al., 2011). Besides, the rapid viscosity analyzer (RVA) profile can distinguish the eating quality for rice varieties with similar AACs by evaluating breakdown viscosity (BDV), setback viscosity (SBV), and consistency viscosity (CSV) (Bao, 2007). Therefore, RVA is always used to evaluate the rice quality. In accordance with Chinese national standard GB/T 17891–1999 (high-quality paddy), the threshold of high-quality glutinous rice was AC ≤2.0% and GC ≥100 mm, with no requirement of GT. However, AC, GC, and GT were required together in the Ministry of Agriculture of China standard NY/T 593-2013.

Tremendous efforts have been made to understand the genetic basis of rice ECQs. There are some related studies focused on SSRGs on how to regulate ECQs and it was shown that there were certain interaction effects between SSRGs. Each enzyme and its gene plays a distinct role (Ball et al., 1996; Myers, 2000; Nakamura, 2002). *ISA1, SBE1*, and *BEIIb* had significant effects on SBV (Xu, 2008). *Soluble starch synthase IIIa* (*SSIIIa*) affected the sB2–B4 chains of starch and affected the RVA profile characteristics and the expression of *Wx* and *SSI* (Fujita et al., 2007). There was also an interaction between *Wx* and *SSIIIa*, and it had a significant influence on all ECQs and RVA profile parameters, except for GT (Yang et al., 2018). The SSRGs cooperated to form a regulatory network that controlled the AAC, alkali spreading value (ASV), RVA profile characteristics, and GC (Tian et al., 2009; Yan et al., 2011).

Furthermore, the effects of *SSIVb* and its interaction with *AGPlar* and pullulanase (*PUL*) are vital for rice quality breeding under the background of the same major genes (Xu et al., 2020). Many studies favored understanding the genetic basis of rice qualities by quantitative trait loci (QTLs) mapping (Leng et al., 2014). Moreover, association analysis is a powerful tool for studying genetic loci involved in the inheritance of complex traits, and it has been successfully exploited in plant molecular genetics (Wang et al., 2019). At present, with the rapid development of bioinformatics and the development of a large number of single nucleotide polymorphisms (SNPs) markers, the research of rice genomics has shifted from qualitative traits to quantitative traits. Nowadays, analyzing quantitative traits genes through association analysis is an essential subject in plant genomics research. Tremendous efforts have been made to understand the genetic regulation of rice quality. Given that starch comprises ~90% of the rice grain (Vandeputte and Delcour, 2004), naturally biosynthesis-related genes involved in starch affect the quality of rice. Targeted-gene association analysis (TGAS), also known as candidate-gene association mapping, relates polymorphisms in selected candidate genes based on prior knowledge from the biochemical pathway of the specific traits of interest. Therefore, TGAS is a powerful tool to resolve complex trait variations such as quality. Although the SSRGs were well-studied, the relationship between the effects of SSRGs polymorphism on glutinous rice quality remains unclear. Sixty-three glutinous rice accessions were used to identify the genotypes of 17 SSRGs and determine their physicochemical indices, RVA profile characteristics, thermal characteristics, and retrogradation characteristics. Simultaneously, 41 pairs of simple sequence repeat (SSR) primers were used to analyze the population structure to exclude false associations. In order to gain a deeper understanding of the functions of these SSRGs on glutinous quality, TGAS was used to clarify the influence of each gene of SSRGs and its interaction on the quality of glutinous rice. The results will provide a theoretical basis for the breeding quality of glutinous rice.

## Materials and Methods

### Plant Materials

A total of 63 glutinous rice accessions (Supplementary Table 1) were grown in an experimental field of the Southwest University of Science and Technology in the rice-growing season with the same conventional cultivating method, and in a randomized block design with one replication. In the replicate, there was four rows and ten plants per row for each accession and they were planted at a distance of inter-row 27 cm and inter-plant 16 cm. Three single plants were, respectively, harvested from each accession at the full-ripe stage (biological replicates). After being air-dried and stored at room temperature for 3 months, all rice samples were dehulled to brown rice, then milled into white rice, afterward ground and passed through a 100-mesh sieve to obtain milled grain flour. All samples were stored in cooling rooms at 4°C until use.

### Measurement of Physical and Chemical Indices of Starch

Polished rice was milled into powder according to the protocol of Xiang et al. (2017). AAC and GC were measured according to the Chinese national standards, GB/T 15683–1995, and standard of Chinese Ministry of Agriculture, GB/T 17891–1999, respectively. GT was measured using differential scanning calorimetry (DSC) according to previously reported methods (Xu et al., 2019). To measure the percent of retrogradation (R%), the gelatinized starch was stored at 4°C for 7 days. Afterward, the starch was equilibrated at room temperature for 1 h with the same thermal program as that of the measurement of GT. The following formula calculated R%:

R% = ΔHr (enthalpy of retrogradation)^*^100%/ΔHg (enthalpy of gelatinization)

Each index per sample was measured in duplicate (parallel test).

### Determination of RVA Profile Characteristics

An RVA (Model No. RVA4500, NewPortSci. Co. Warriewood, Australia) was used to assess RVA profile characteristics following the instructions of the manufacturer and according to the standard method of the American Association of Cereal Chemists: AACC61-02. Three original parameters were obtained from the RVA profile, namely: peak viscosity (PKV), hot paste viscosity (HPV), cool paste viscosity (CPV), and the three secondary parameters obtained, BDV = PKV-HPV, SBV = CPV-PKV, CSV = CPV-HPV, were used to evaluate rice starch viscosity characteristics. In addition, rice starch viscosity characteristics also included pasting temperature (PaT) and peak time (PeT). Each starch sample was repeated two times.

### Phenotypic Data Analysis

The statistical analyses of phenotypic data such as Pearson's correlation, ANOVA, multiple comparisons (Tukey's test), and so on were performed using the SPSS Statistics 19 software (IBM Institute Inc., Armonk, NY, USA) and Excel 2016.

### DNA Extraction and Gene Genotyping

About 100 mg of young leaves at the tillering stage were collected from one plant of each accession and ground using a Fastprep Sample Rapid Crushing System (MP Biomedicals, Santa Ana, CA, USA). DNA was extracted as described by Xiang et al. (2007).

In order to eliminate the influence of the population structure on the analysis and prevent “false linkage,” 41 pairs of SSR markers were selected as per the method of Yan et al. (2011), and 17 SSRGs were selected as target genes, including *AGPlar, AGPsma, AGPiso, GBSSII, SBE1, BEIIb, BEIIa, PUL, ISA1, SSI, SSIIc, SSIIb, SSIIa, SSIIIb, SSIIIa, SSIVa*, and *SSIVb*. They were used to identify the genotypes of SSRGs alleles employing the primers designed according to Yan et al. (2011). Detailed primer information was shown in Supplementary Table 2.

PCRs were carried out using an Eppendorf Thermal Cycler (Mastercycler® nexus GSX1, Germany) with a total of 15 μL reaction mixtures and the Golden Easy PCR System (TIANGEN, Beijing, China). All of the primers were synthesized by Sangon Biotech (Shanghai, China). A total of 5 μL of each PCR product of CAPS markers was digested with the corresponding restriction endonuclease in a reaction mixture with a total volume of 15 μL, which included 1.5 μL of 10 × buffer, five units of restriction endonuclease, and sterile molecular biology grade water. Later, the reaction was performed at 37°C for 3–3.5 h. All the amplified products were detected on 3% agarose gel in a 0.5 × Tris-Borate EDTA (TBE) buffer using GreenView (Applied BioProbes, Rockville, MD, USA).

### Genotypic Data and Population Structure Analysis

The PowerMarker (version 3.25) software was used to calculate the polymorphism information content (PIC) of each marker. The neighbor-joining tree was built based on Nei's genetic distance according to the results of PCR by amplifying 41 SSR primers and framed using the MEGA 5.0 software by the unweighted pair-group method with arithmetic means (UPGMA). The population structure of 63 accessions was analyzed by the STRUCTURE 2.3 software with the Bayesian clustering method (Tang et al., 1991; Li et al., 2004).

### Targeted-Gene Association Analysis

Targeted-gene association analysis was conducted on 46 molecular markers of the target genes identified from the SSRGs and the mean values of 16 measured parameters of starch traits from 63 rice accessions. In order to control false positives, the K (the kinship matrix) + Q (population structure matrix) model was used for TGAS based on the mixed linear model (MLM) in the TASSEL 2.3 software, and the detection of marker-trait association was determined by the *P*-value (marker) (*P* < 0.05). The Q matrix was obtained from the results of STRUCTURE 2.3.4 software analysis. The K matrix was generated from the results of relatedness analysis using the kinship matrix function in TASSEL 2.3 (Wang et al., 2019). The most probable number for the subpopulation was chosen according to ΔK, which is an *ad hoc* quantity related to the log probability of data with respect to the number of clusters inferred by the Structure. The significance of the marker was determined by the *P*-value (*P* < 0.05) of the F test of ANOVA. Then, significant markers were, respectively, used for multi-factor variance analysis and for determining the significance of gene interaction (*P* < 0.05), which were performed with aov() function of R software (https://www.r-project.org/).

## Results

### Phenotypic Evaluations

The AAC of all materials was <2%, which was consistent with the typical characteristics of glutinous rice. The detailed phenotypic data of each material were listed in Supplementary Tables 3–5. The results clearly showed that materials significantly differed in most quality characteristics (Table 1). Most of the pasting viscosity parameters (PVP) and all the retrogradation properties (RP), such as PKV, HPV, CPV, BDV, SBV, CSV, enthalpy of retrogradation (ΔHr), and retrogradation percentage (R%), varied widely among these materials. The variations of CSV, ΔHr, and R% were 49.05, 48.66, and 41.98%, respectively. In contrast, the variations of GC, a fraction of RVA profiles, and the thermal characteristics were relatively narrow. These results showed that the most of quality characters were suitable for the steps following genetic analysis.

**Table 1:** The phenotypic data of different glutinous rice germplasms.

**Traits**	**Mean ± SE**	**Maximum**	**Minimum**	**CV (%)**
AAC (%)	0.11 ± 0.04	1.88	0.00	254.65
GC (cm)	10.24 ± 0.09	11.83	7.13	7.06
PKV (cp)	2,261.16 ± 61.61	3,524.00	1,190.50	21.63
HPV (cp)	953.12 ± 38.38	1,853.50	301.00	31.96
CPV (cp)	1,276.87 ± 56.09	3,165.00	427.67	34.87
BDV (cp)	1,308.05 ± 33.60	2,057.00	839.50	20.39
SBV (cp)	−984.29 ± 30.75	−359.00	−1,684.50	−24.79
CSV (cp)	323.76 ± 20.01	1,311.50	126.70	49.05
PeT (min)	3.82 ± 0.04	4.58	3.37	8.44
PaT (°C)	73.87 ± 0.49	82.33	68.95	5.24
To (°C)	67.61 ± 0.57	77.57	62.02	6.73
Tp (°C)	73.51 ± 0.50	82.12	68.97	5.41
Tc (°C)	81.33 ± 0.40	87.37	76.99	3.90
ΔT1/2 (°C)	7.92 ± 0.15	10.11	5.50	15.37
ΔHg (J/g)	10.59 ± 0.13	13.26	7.70	9.78
ΔHr (J/g)	3.31 ± 0.20	6.99	0.70	48.63
R%	0.31 ± 0.02	0.62	0.09	41.97

*AAC, apparent amylose content; GC, gel consistency; PKV, peak viscosity; HPV, hot paste viscosity; CPV, cool paste viscosity; BDV, breakdown viscosity; SBV, setback viscosity; CSV, consistence viscosity; PeT, peak time; PaT, pasting temperature; To, onset temperature; Tp, peak temperature; Tc, conclusion temperature; ΔT1/2, width at half peak height; ΔHg, enthalpy of gelatinization; ΔHr, enthalpy of retrogradation; R%, percentage of retrogradation; SE, the standard deviation; CV, coefficient of variation. Same below*.

### Correlation Analysis on Physicochemical Properties

A pairwise correlation analysis was carried out to investigate the relationships among the tested physicochemical properties in 63 glutinous rice materials. According to Table 2, the correlations between GC and HPV, CPV, PaT, PeT, onset temperature (To), peak temperature (Tp), conclusion temperature (Tc), ΔHr, and R% were significantly negative (*P* < 0.05 or *P* < 0.01). However, the correlations between most of the PVP and the thermal properties were significantly positive (*P* < 0.05 or 0.01), except for ΔT1/2.

**Table 2:** Correlations among PVP, thermal and RP, and GC.

	**PKV**	**HPV**	**CPV**	**BDV**	**SBV**	**CSV**	**PeT**	**PaT**	**GC**
To	0.273^*^	0.555^**^	0.500^**^	−0.134	0.366^**^	0.338^**^	0.912^**^	0.980^**^	−0.449^**^
Tp	0.243	0.544^**^	0.479^**^	−0.176	0.387^**^	0.299^*^	0.938^**^	0.983^**^	−0.452^**^
Tc	0.301^*^	0.639^**^	0.624^**^	−0.177	0.534^**^	0.524^**^	0.923^**^	0.927^**^	−0.419^**^
ΔT1/2	−0.342^**^	−0.486^**^	−0.498^**^	−0.072	−0.223	−0.463^**^	−0.594^**^	−0.756^**^	0.373^**^
ΔHg	0.184	0.300^*^	0.212	−0.005	0.018	0.020	0.594^**^	0.617^**^	−0.210
ΔHr	0.345^**^	0.544^**^	0.460^**^	0.011	0.147	0.246	0.812^**^	0.856^**^	−0.357^**^
R%	0.347^**^	0.547^**^	0.459^**^	0.012	0.141	0.237	0.789^**^	0.823^**^	−0.370^**^
GC	−0.227	−0.313^*^	−0.295^*^	−0.058	−0.084	−0.227	−0.449^**^	−0.461^**^	

^*^ and ^**^ indicate significance at P ≤ 0.05 (two-tail) and P ≤ 0.01 (two-tail), respectively.

### Population Genetic Structure Analysis

**Figure 1:**
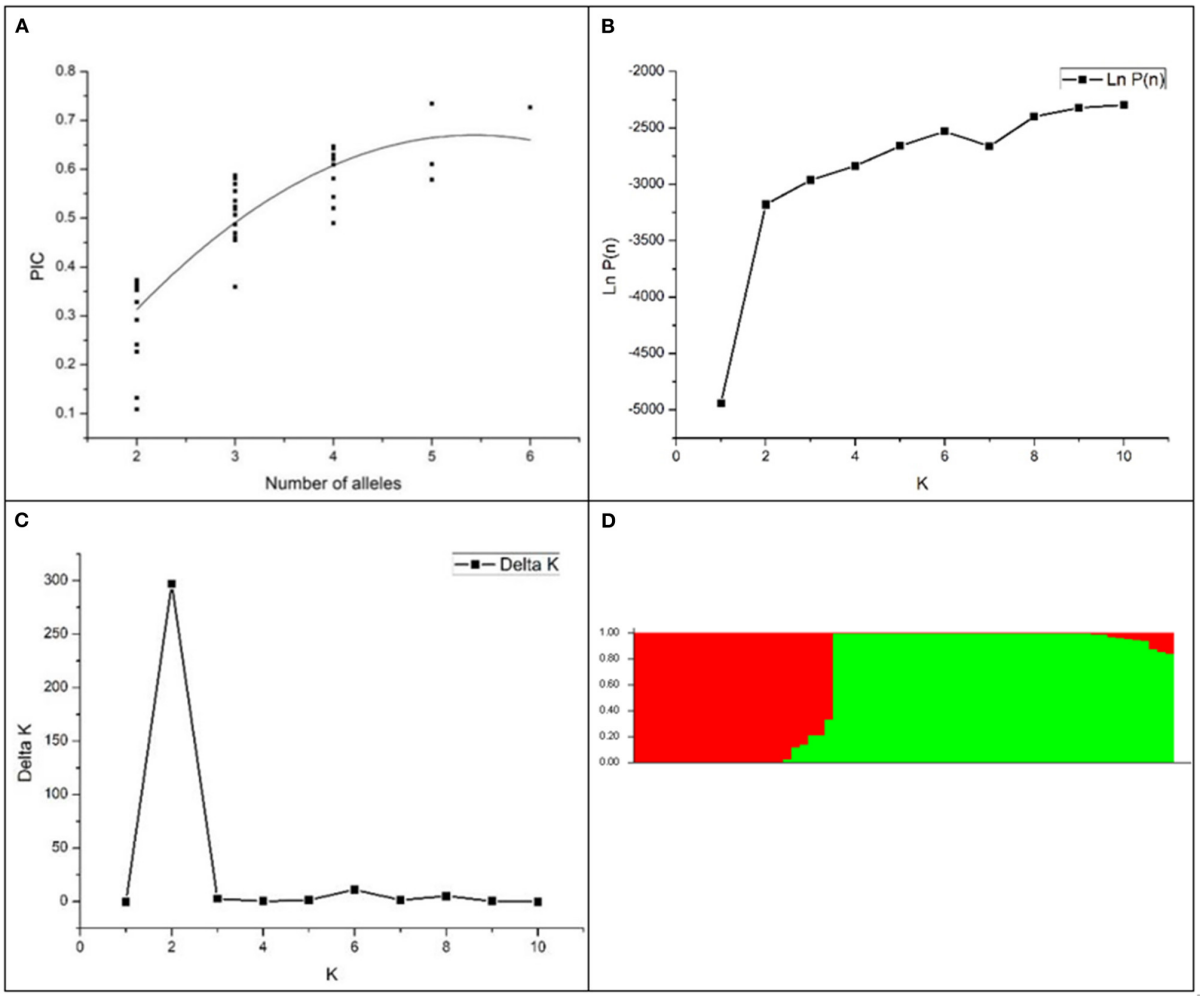
The population structure and divergence of 63 rice cultivars. **(A)** a scatter plot of PIC vs. the number of alleles per SSR locus; **(B)** the mean Ln*P*(D) changed with the number of subgroups; **(C)** the ΔK value changed with the number of subgroups; **(D)** population structure distribution of 63 glutinous accessions. Red, the *japonica* group; green, the *indica* group.

The genetic diversity of the 63 accessions was analyzed using 41 SSR markers, and a total of 129 amplified polymorphic bands were detected. The number of alleles per locus ranged from 2 to 8 (RM528), with an average of 3.1 alleles per locus (Table 3). The average value of PIC was 0.47 and ranged from 0.11 (RM308) to 0.73 (RM251). There were 21 highly polymorphic sites (PIC >0.5) and 16 moderately polymorphic sites (0.25 < PIC < 0.5). The results indicated rich polymorphism for the selected RM primers in the glutinous accessions and can be used for group structure analysis. Generally, the more abundant the allelic variation, the higher the PIC. Correlation analysis showed that there was a significant positive correlation between the allelic variation and PIC of the labeled locus in this study (*P* < 0.01). Non-linear regression analysis showed a linear relationship between the number of allelic variance and PIC (Figure 1A).

**Table 3:** The genetic diversity of SSR markers in glutinous rice.

**Marker**	**Allele number**	**PIC**	**Marker**	**Allele number**	**PIC**	**Marker**	**Allele number**	**PIC**
RM5	4	0.5464	RM251	5	0.7330	RM412	3	0.4270
RM11	3	0.5333	RM252	5	0.5735	RM471	2	0.2467
RM14	5	0.5869	RM255	3	0.5078	RM474	4	0.6371
RM17	3	0.5404	RM258	3	0.5267	RM475	4	0.6322
RM101	4	0.4584	RM259	2	0.3071	RM489	2	0.3561
RM122	2	0.3641	RM263	4	0.5872	RM519	3	0.3421
RM128	3	0.4700	RM264	4	0.6159	RM520	2	0.3604
RM180	2	0.2970	RM286	4	0.5918	RM525	3	0.4676
RM205	2	0.3457	RM289	3	0.4519	RM528	8	0.7142
RM211	2	0.3698	RM308	2	0.1119	RM566	2	0.3641
RM216	2	0.2314	RM316	3	0.5212	RM585	4	0.6171
RM223	3	0.5816	RM332	2	0.1355	RM587	3	0.5863
RM234	2	0.3698	RM335	3	0.5743	OSR28	2	0.3512
RM242	3	0.5724	RM336	4	0.5288	Mean	3.1	0.4667

*PIC, polymorphism information content*.

**Figure 2:**
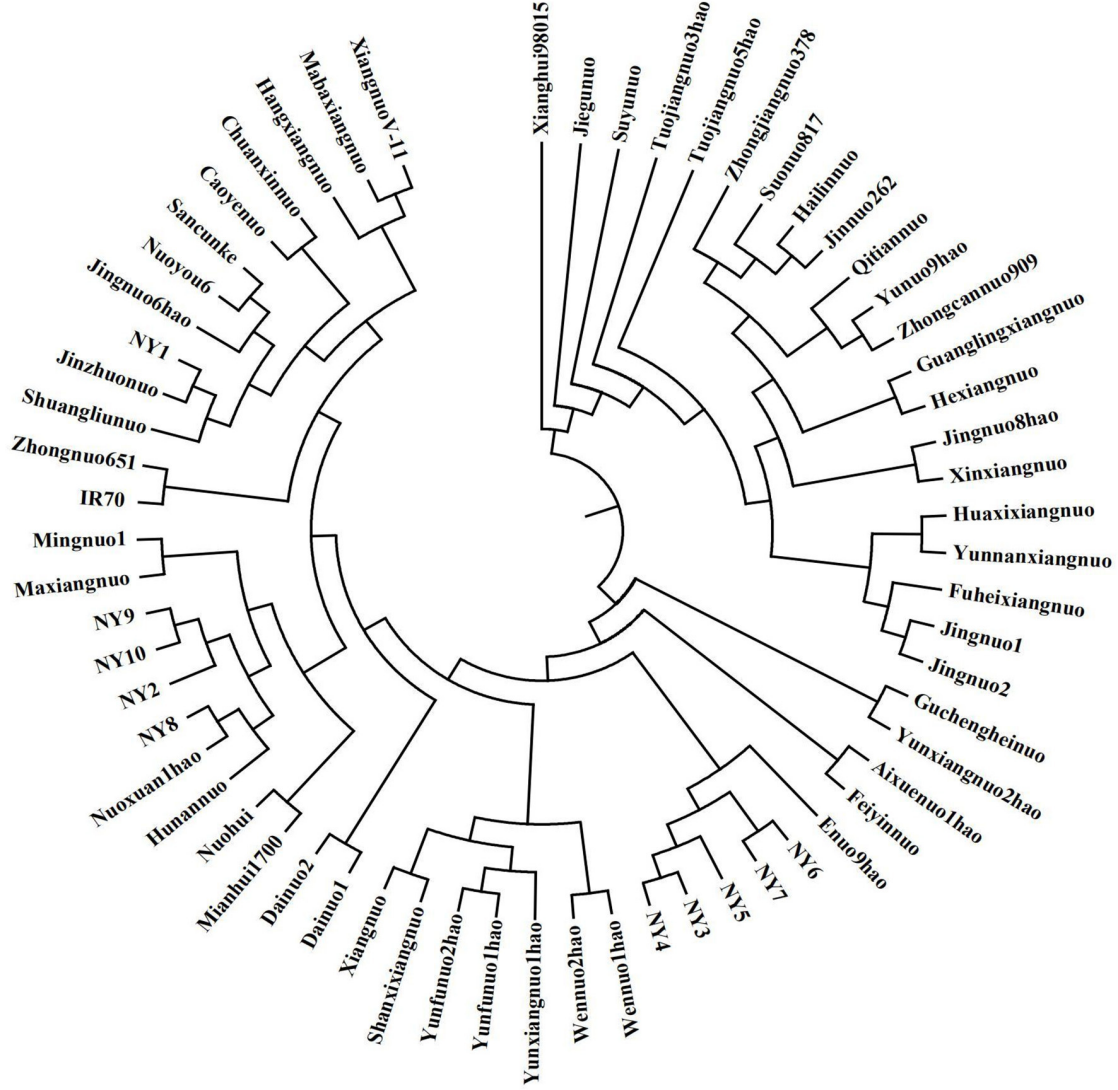
A dendrogram of rice landraces based on Nei's genetic distance.

An analysis of the model-based population structure provided evidence for a significant population structure among the 63 rice accessions. The log-likelihood values increased along with the model parameter K (Figure 1B); thus, ΔK as the diagnostic criterion was used to determine a suitable value for K. The highest ΔK value was obtained at *K* = 2 (Figure 1C). The neighbor-joining tree based on Nei's genetic distances showed that the population was divided into two subpopulations (Figure 2). One group was *japonica* and the other group was *indica*. The same result was seen according to the structural diagram of the test materials (Figure 1D). The results showed that most of the cultivars had a relatively pure genetic background. However, some of the materials were mixed type, which may come from the interspecific hybridization of *indica* and *japonica* subspecies during the long-term domestication of rice. Furthermore, to calculate the Q-value of each individual with *K* = 2, which was the probability of each individual being classified into each subgroup. The *Q*-value of each glutinous accession was used as a covariate in the association analysis between the markers and the phenotypic traits (Supplementary Table 6).

### Inheritance and Gene Interactions of Glutinous Quality

Detailed results of genotyping were listed in Supplementary Table 7. The TGAS showed that many genes were involved in regulating glutinous quality (Table 4). Among them, *SSI, SSIIa, BEIIa*, and *PUL* had significant genetic effects on the glutinous quality of rice (*P* < 0.01). Most quality traits were affected by at least two genes except ΔT1/2 and ΔHg, which were only regulated by *SSIIa* and *SSI*, respectively, especially the GC being regulated by eight genes. The analysis on the interactions between SSRGs indicated that there were significant interactions on glutinous quality traits except for SBV, ΔT1/2, and ΔHg (Table 5). It showed that genetic mechanism of glutinous rice was complicated.

**Table 4:** The results of TGAS of glutinous rice quality.

**Trait**	**Gene**	***F*-value**	***P*-value**	**Trait**	**Gene**	***F*-value**	***P*-value**
GC	*SBE1*	7.2859	0.0091	CSV	*SSIIa*	8.2303	0.0057
	*SSIIa*	7.9807	0.0064		*PUL*	15.0005	0.0002
	*BEIIa*	8.0661	0.0062	PeT	*SSI*	13.0238	0.0006
	*PUL*	8.5797	0.0048		*SSIIa*	16.0739	0.0001
	*SSIIc*	10.5139	0.0019	PaT	*SSI*	12.6373	0.0007
	*BEIIb*	12.2691	0.0008		*SSIIa*	22.3431	1.50E-05
	*SSIVb*	14.9373	0.0002	To	*SSI*	11.2827	0.0014
	*ISA1*	16.1881	0.0001		*SSIIa*	23.5643	9.50E-06
PKV	*BEIIa*	9.0398	0.0039	Tp	*SSI*	12.6219	0.0007
	*PUL*	14.4515	0.0003		*SSIIa*	21.6839	1.92E-05
HPV	*SSIIa*	9.9417	0.0025	Tc	*SSI*	16.1789	0.0001
	*PUL*	13.2807	0.0005		*SSIIa*	16.9379	0.0001
CPV	*SSIIa*	9.7383	0.0028	ΔT1/2	*SSIIa*	18.9986	5.42E-05
	*PUL*	14.0856	0.0004	ΔHg	*SSI*	12.1011	0.0009
BDV	*PUL*	6.9954	0.0014	ΔHr	*SSI*	9.7726	0.0027
	*SSIVa*	8.3119	0.0055		*SSIIa*	23.9928	8.11E-06
	*BEIIa*	11.1981	0.0014	R%	*SSI*	7.4215	0.0085
SBV	*SSIVa*	8.4388	0.0052		*SSIIa*	29.019	1.36E-06
	*BEIIb*	7.717	0.0073				

**Table 5:** The interaction effects of SSRGs in glutinous rice.

**Trait**	**Gene**	***F*-value**	***P*-value**	**Trait**	**Gene**	***F*-value**	***P*-value**
GC	*ISA1* × *SSIVb*	9.234	0.0083	CSV	*SSIIa* × *PUL*	0.937	0.0381
	*ISA1* × *PUL*	5.327	0.0145	PeT	*SSIIa* × *SSI*	6.043	0.0396
	*SSIVb* × *PUL*	5.359	0.0006	PaT	*SSIIa* × *SSI*	6.875	0.0432
	*BEIIb* × *PUL*	3.016	0.0243	To	*SSIIa* × *SSI*	32.143	0.0048
	*SSIIa* × *BEIIb*	9.717	0.0356	Tp	*SSIIa* × *SSI*	12.602	0.0238
PKV	*BEIIa* × *PUL*	2.514	0.0496	Tc	*SSIIa* × *SSI*	49.146	0.0022
HPV	*SSIIa* × *PUL*	5.677	0.0021	ΔHr	*SSIIa* × *SSI*	9.613	0.0362
CPV	*SSIIa* × *PUL*	6.532	0.0009	R%	*SSIIa* × *SSI*	23.573	0.0083
BDV	*BEIIa* × *PUL*	9.953	0.0351				
	*SSIVa* × *PUL*	8.652	0.0434				

**Figure 3:**
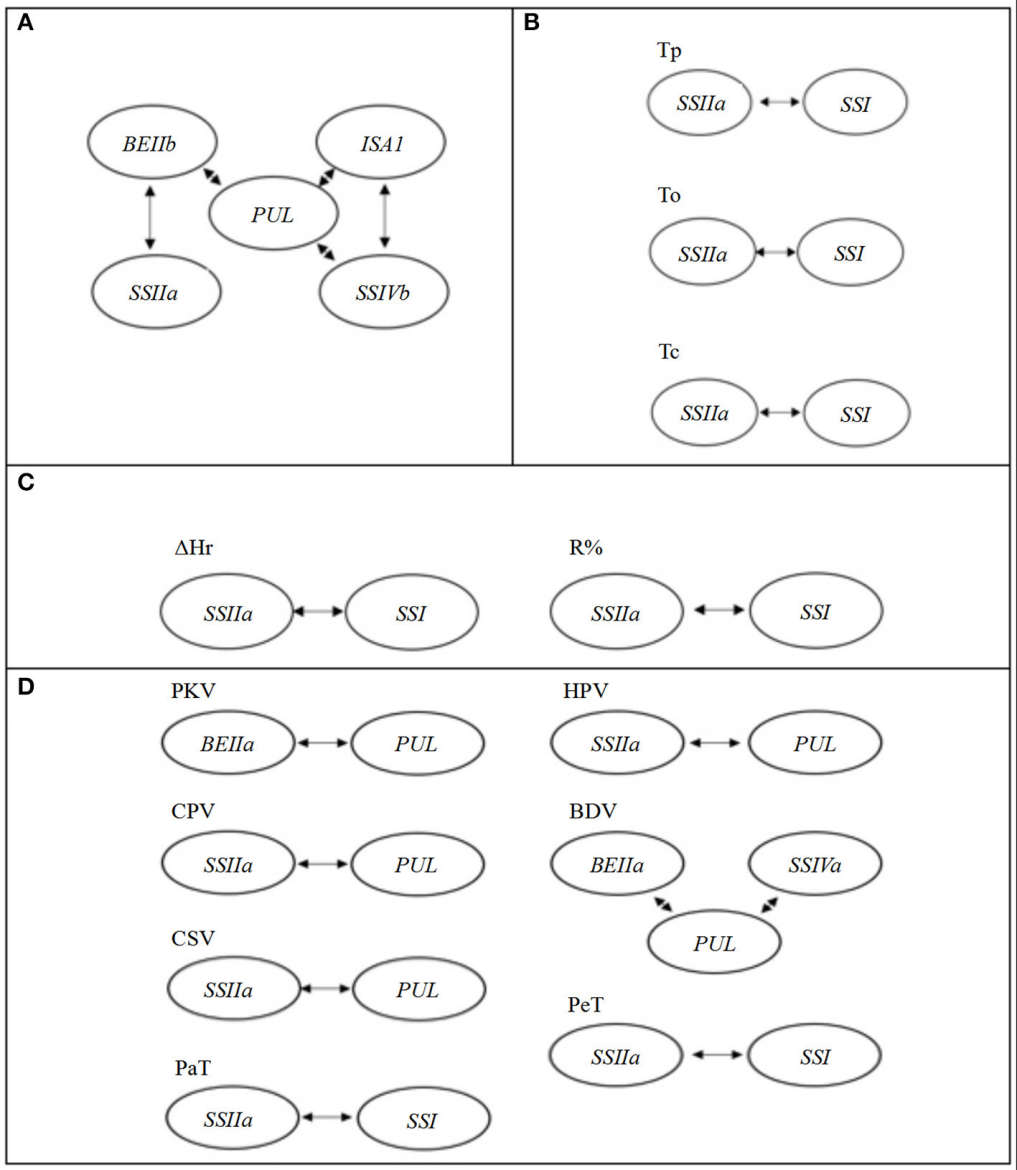
The gene interaction effects for different quality traits. **(A)** The starch gel consistency (GC); **(B)** the starch thermal properties; **(C)** the starch RP; **(D)** the starch pasting viscosity parameters.

The TGAS results showed that eight genes of 17 SSRGs were involved in the genetic regulation of GC, including *SSIIc, SSIIa, SSIVb, SBE1, BEIIb, BEIIa, ISA1*, and *PUL* (Table 4). Moreover, their genetic effects were significant (*P* < 0.01). There were significant interactions between *PUL* and *SSIIa, SSIVb, BEIIb, ISA1* (*P* < 0.05 or 0.01). *PUL* was central in the regulation of GC, and it had the most significant impact (Table 5 and Figure 3A).

The results of TGAS and gene interactions of RP were very similar to those of starch thermal properties. All the traits were associated with both *SSI* and *SSIIa* except for ΔT1/2 and ΔHg (Table 4), and the interaction between *SSIIa* and *SSI* existed in all traits except for ΔT1/2 and ΔHg (Table 5 and Figures 3B,C). *SS IIa* and *SSI* were the major genes that regulated thermal properties and RP.

The genetic mechanism of PVP was complicated, and TGAS showed that it involved six SSRGs. All of the traits were associated with *PUL* or *SSIIa* except for SBV, PeT, Pet, PKV, and BDV. In addition to these two common genes, PKV was associated with *BEIIa;* BDV was associated with *SSIVa and BEIIa*; PeT and PaT were associated with *SSI*; and SBV was associated with *SSIVa* and *BEIIb* (Table 4). Complex genetic interactions were found in PVP except for SBV. The interaction between *BEIIa* and *PUL* was identified to regulate PKV, and the interaction between *PUL* and *SSIIa* was also found to affect HPV, CPV, and CSV. Moreover, the interactions between *PUL, BEIIa*, and *SSIVa* significantly affected BDV, and the interaction between *SSIIa* and *SSI* significantly regulated PeT and PaT (*P* < 0.05, Table 5 and Figure 3D).

## Discussion

The biosynthesis of starch is complex, and the genes involved in starch synthesis have specific functions and complex gene interactions. The epistasis of *Wx* gene over the other SSRGs is eliminated in glutinous rice, and the increased effects of genes in amylopectin synthesis can be studied compared to non-glutinous rice. TGAS will illuminate the effects of SSRGs on phenotypic traits. Given that linkage disequilibrium may be caused by the mixing of subpopulations, which could lead to false positives if it is not correctly controlled in the statistical analysis, an estimate of the population structure must be made in the association analysis (Yu and Buckler, 2006). In this study, 63 rice accessions were classified into two subpopulations, *indica* and *japonica*, by 41 pairs of SSR primers and a method of model-based population structure (Figure 1D), and a dendrogram was created based on Nei's genetic distances (Figure 2). Simultaneously, the materials in this study significantly differed on a majority of quality characters (Table 1), and the correlations among quality traits are significant (*P* < 0.05 or 0.01, Table 2). There was polymorphism for the selected RM primers in the materials, and the PIC varies from 0.1088 to 0.7330 (Table 3). These results provided a solid foundation for TGAS.

As in earlier studies, we found that *SBE1, BEIIb, SSIVb*, and *PUL* affected GC (He et al., 2006; Tian et al., 2009; Xu et al., 2020). Besides, *ISA1, SSIIc, BEIIa*, and *SSIIa* were also associated with GC, and their genetic effects decreased in order (Table 4). However, these genes are not related to the regulation of GC in previous studies. The *PUL* was at the center of gene interaction in GC regulation, and certain genes affected GC by way of gene interactions (Figure 3A).

Targeted-gene association analysis showed that *SSIIa* and *SSI* played essential roles in controlling thermal and RP (Table 4). The result was in agreement with previous findings (Bao et al., 2006; Xu et al., 2013). Gene interaction between *SSIIa* and *SSI* existed widely in thermal properties and RP except for ΔT1/2 and ΔHr (Table 5 and Figures 3B,C). Related studies have shown that both *SSIIa* and *SSI* can elongate the short chains of amylopectin, resulting in a higher GT (Umemoto et al., 2002; Fujita et al., 2006). These results showed that *SSIIa* and *SSI* were of great significance in glutinous rice quality.

Our results indicated that six of the 17 SSRGs were associated with PVP. The *PUL* gene played a crucial role in controlling PKV, HPV, CPV, BDV, SBV, and CSV in glutinous rice (Table 4). This result is similar to Yan et al. (2011) but contrary to Xu et al. (2013). The reason may be the difference in glutinous rice accessions that were used in the TGAS. Li et al. (2017) stated that importance of a marker on certain phenotypic traits could be overestimated when samples with a narrow genetic base are used for association analyses. Yan et al. (2011) used more *japonica* (78) than *indica* (20) and also included some *javanica* (4), intermediate rice (8), and African cultivated rice, *O. glaberrima* (8); Xu et al. (2013) used 28 *indica* and 22 *japonica*; the present materials comprised of 42 *indica* and 21 *japonica*. The number of samples and their relative balance between *indica* and *japonica* in TGAS may affect the results. Also, the method for measuring PKV was different, and the method of Yan et al. (2011) was used in this study, but not that of Xu et al. (2013). Equally important, different primers were used to evaluate the population structure. Both this study and Yan et al. (2011) used SSR primers, while Xu et al. (2013) used the primers of amplified fragment length polymorphism (AFLP) and inter-simple sequence repeat (ISSR). The population structure can result in misleading signals that mimic genuine association (Teo, 2008). SSR has the advantages of stability, high polymorphism, and simple operation, which may provide more reliable results for population structure analysis. Meanwhile, the *PUL* gene participated in the interactions of PVP except for PeT and PaT, and it regulated most of the PVP in glutinous rice (Table 5 and Figure 3D).

**Figure 4:**
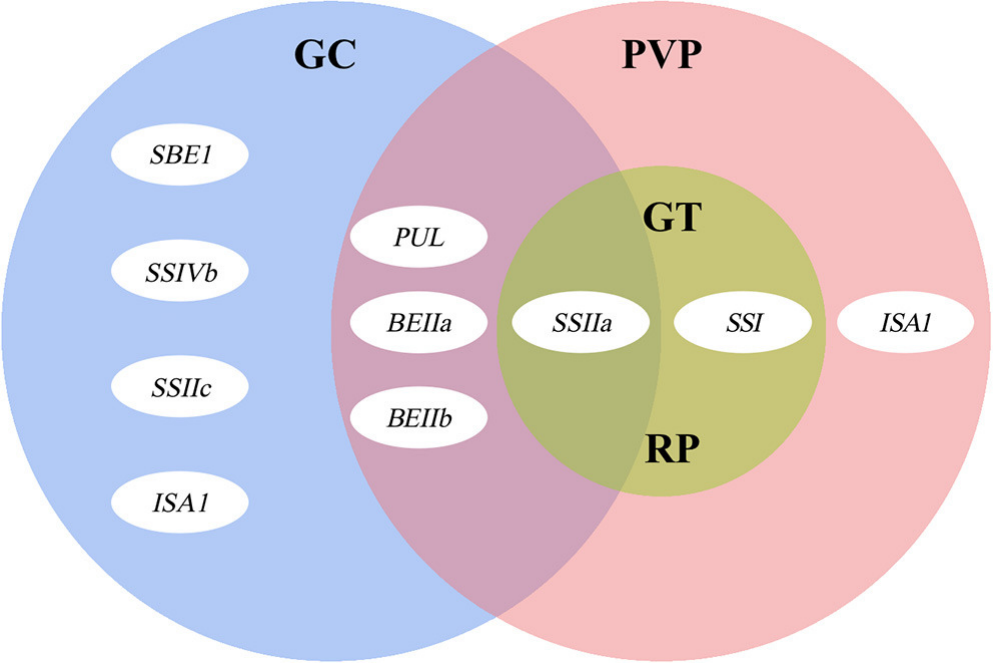
The summary of genes controlling rice grain ECQs. The three large ovals represent gel consistency (GC, blue), pasting viscosity parameters (PVP, pink), gelatinization temperature (GT, green), and retrogradation properties (RP, green), and a small oval proportionally represents the role of each gene.

These results strongly suggest that SSRGs regulating GC, PVP, RP, and GT of glutinous rice, form a fine network, and the *SSIIa* gene are central in determining grain ECQs affecting all four properties: GC, PVP, RP, and GT (Figure 4). *SSIIa* regulates GT and RP as a major gene and affects GC and PVP as a minor gene, which is associated with all indicators except for PKV, BDV, and SBV (Table 4). Besides, we found that multiple genes involve interactions with *SSIIa* in quality traits (Table 5), which further shows that *SSIIa* is the major gene in determining the ECQs of glutinous rice. The role of *SSIIa* in regulating glutinous rice quality may be the same as that of *Wx* in non-glutinous rice.

Overall, we described a strategy to clarify the influence of each of the SSRGs and their interaction on glutinous rice quality. Vast characteristic differences exist in the physicochemical properties of starch in glutinous rice accessions, especially in the PVP and RP. *SSIIa, SSI*, and *PUL* mainly control most starch properties in glutinous rice, but *SSIIa* is central among them. Furthermore, the effects of *BEIIb, ISA1, SSIVb, BEIIa*, and *SSIVa* and their interactions with the major genes also affect starch properties, especially in GT and PVP. The findings provide a more comprehensive understanding of the effects of SSRGs in regulating glutinous rice quality and illustrate the possible gene interactions.

## Data Availability Statement

The datasets presented in this study can be found in online repositories. The names of the repository/repositories and accession number(s) can be found in the article/Supplementary Material.

## Author Contributions

OZ, CL, and BY investigated the genetic studies. BY, HY, and LX carried out the analysis of physical and chemical parameters. YC collected and planted the materials. XX designed the overall project. OZ and CL analyzed the data and wrote the manuscript. All authors have read and approved the final manuscript.

## Conflict of Interest

The authors declare that the research was conducted in the absence of any commercial or financial relationships that could be construed as a potential conflict of interest.

## Publisher's Note

All claims expressed in this article are solely those of the authors and do not necessarily represent those of their affiliated organizations, or those of the publisher, the editors and the reviewers. Any product that may be evaluated in this article, or claim that may be made by its manufacturer, is not guaranteed or endorsed by the publisher.
