# Supplementary material for: Effects of Starch Synthesis-Related Genes Polymorphism on Quality of Glutinous Rice
Source: Front Plant Sci. 2021 Aug 6;12:707992. doi: 10.3389/fpls.2021.707992 (PMC8377722; doi:10.3389/fpls.2021.707992)
Supplement: Supplementary file 1 [file Data_Sheet_1.doc]

**Supplementary Materials**

**Supplementary Table 1** **|** List of 63 glutinous accessions used in this study.

**Supplementary Table 2 |** Primer sequences of molecular markers used to identify the genotypes of 17 SSRGs in rice.

**Supplementary Table 3 |** The results of gel consistency and apparent amylose content for 63 glutinous accessions.

**Supplementary Table 4 |** The results of RVA profile characteristics for 63 glutinous accessions.

**Supplementary Table 5 |** The results of thermal and retrogradation properties for 63 glutinous accessions.

**Supplementary Table 6 |** The Q-values of 63 glutinous accessions.

**Supplementary Table 7 |** The results of gene genotyping for 63 glutinous accessions.

Supplementary Materials

**Table S1 |** List of 63 glutinous accessions used in this study.

| Number | Accession | Type | Number | Accession | Type |
| --- | --- | --- | --- | --- | --- |
| 1 | Guchengheinuo | *Indica* | 33 | Zhongcannuo909 | *Japonica* |
| 2 | Jingnuo8hao | *Japonica* | 34 | Yunuo9hao | *Japonica* |
| 3 | Jingnuo6hao | *Indica* | 35 | Xinxiangnuo | *Japonica* |
| 4 | Aixuenuo1hao | *Indica* | 36 | Hangxiangnuo | *Indica* |
| 5 | Hexiangnuo | *Japonica* | 37 | Enuo9hao | *Indica* |
| 6 | Mabaxiangnuo | *Indica* | 38 | Dainuo1 | *Indica* |
| 7 | Huaxixiangnuo | *Japonica* | 39 | Dainuo2 | *Indica* |
| 8 | Jiegunuo | *Japonica* | 40 | Jingnuo1 | *Japonica* |
| 9 | Chuanxinnuo | *Indica* | 41 | Jingnuo2 | *Japonica* |
| 10 | Guanglingxiangnuo | *Japonica* | 42 | Maxiangnuo | *Indica* |
| 11 | Feiyinnuo | *Indica* | 43 | Jinzhuonuo | *Indica* |
| 12 | Fuheixiangnuo | *Japonica* | 44 | Mingnuo1 | *Indica* |
| 13 | IR70 | *Indica* | 45 | Hunannuo | *Indica* |
| 14 | Mianhui1700 | *Indica* | 46 | NY1 | *Indica* |
| 15 | Nuoxuan1hao | *Indica* | 47 | NY2 | *Indica* |
| 16 | Nuohui | *Indica* | 48 | NY3 | *Indica* |
| 17 | Qitiannuo | *Japonica* | 49 | NY4 | *Indica* |
| 18 | Suyunuo | *Japonica* | 50 | NY5 | *Indica* |
| 19 | Shanxixiangnuo | *Indica* | 51 | NY6 | *Indica* |
| 20 | Tuojiangnuo3hao | *Japonica* | 52 | NY7 | *Indica* |
| 21 | Tuojiangnuo5hao | *Japonica* | 53 | NY8 | *Indica* |
| 22 | Wennuo1hao | *Indica* | 54 | NY9 | *Indica* |
| 23 | Wennuo2hao | *Indica* | 55 | NY10 | *Indica* |
| 24 | Xiangnuo | *Indica* | 56 | Nuoyou6 | *Indica* |
| 25 | XiangnuoV-11 | *Indica* | 57 | Caoyenuo | *Indica* |
| 26 | Xianghui98015 | *Japonica* | 58 | Sancunke | *Indica* |
| 27 | Yunxiangnuo1hao | *Indica* | 59 | Shuangliunuo | *Indica* |
| 28 | Yunxiangnuo2hao | *Indica* | 60 | Hailinnuo | *Japonica* |
| 29 | Yunnanxiangnuo | *Japonica* | 61 | Jinnuo262 | *Japonica* |
| 30 | Yunfunuo1hao | *Indica* | 62 | Zhongjiangnuo378 | *Japonica* |
| 31 | Yunfunuo2hao | *Indica* | 63 | Suonuo817 | *Japonica* |
| 32 | Zhongnuo651 | *Indica* |  |  |  |

**Table S2 |** Primer sequences of molecular markers used to identify the genotypes of 17 SSRGs in glutinous rice.

| Gene name | Marker name | Primer sequence (5′→3′) | Marker type |
| --- | --- | --- | --- |
| *AGPlar* | *AGPlar* M1* | [F] CGTTCAGGTTCAGGCAATCA | STS |
|  |  | [R] GGAAGGGTGGTGATGTGGAG |  |
|  | *AGPlar* M2 | [F] GCGTGAACTGAACATCCATCT | CAPS (*Tsp45 I*^#^) |
|  |  | [R] GGTTCAAGCCTTCAGGTCAG |  |
| *AGPiso* | *AGPiso* M2* | [F] CAATCGCTGCCATCGGTTG | STS |
|  |  | [R] TTCCACATCGTTAGGTACACG |  |
|  | *AGPiso* M3 | [F] TGGAATGGGAACTCTATTATTGG | CAPS (*EcoR I*) |
|  |  | [R] TCCCAACCTCTACCTTCAAATG |  |
| *AGPsma* | *AGPsma* M1* | [F] TCTATTCTCAGCCCTCCAACC | STS |
|  |  | [R] GTGTGTTTAGAGGTGCTTTTCG |  |
|  | *AGPsma* M2 | [F] TACGCTATGCTCTTGAAAC | STS |
|  |  | [R] TATCTTCCCAGTAACCATCA |  |
| *GBSSII* | *GBSSII* M1* | [F] TTGCTGCGAATTATCTGCG | STS |
|  |  | [R] ACCTCCTCCCACTTCTTTGC |  |
| *SSI* | *SSI* M1* | [F] GGTAGGGTAGGTCAATCTGGC | CAPS (*Nru I*) |
|  |  | [R] ATAGAGAAGACAATGTGGCAACC |  |
|  | *SSI* M2* | [F] CTTCTATCCATTCCTTAATCCCA | STS |
|  |  | [R] ATGCTATTGATGTTAAGAGGGC |  |
|  | *SSI* M3 | [F] GACCCACCTCGCTATCTGTTG | CAPS (*Apa I*) |
|  |  | [R] GGAAACACCAGACATCAACCAG |  |
| *SSIIc* | *SSII-1* M1* | [F] CACCCCACCGTTCTACTATGC | STS |
|  |  | [R] TCCATAGTTTCATTGAGATTGCTC |  |
|  | *SSII-1* M2* | [F] CAAGTTGGTGACGATAGTGATGA | CAPS (*Age I*) |
|  |  | [R] AACAGAGCCTCCATTACCTTTAC |  |
|  | *SSII-1* M3 | [F] AGAGATCAAATCGTGGAAC | STS |
|  |  | [R] TGGAGTGAAGTAGTGGAAT |  |
|  | *SSII-1* M4 | [F] ATCTTTAGACGATTAGCG | STS |
|  |  | [R] AAGTCACAAGTAGAAGGG |  |
| *SSIIb* | *SSII-2* M1 | [F] AGATTTGAACTCAGGACTTGGTG | STS |
|  |  | [R] TCTATGGGCTCTATCCTTACTAGG |  |
|  | *SSII-2* M2* | [F] CGCTCGTTGCCTAGCTAGC | STS |
|  |  | [R] GGCGAGGAAGCGATTGCC |  |
|  | *SSII-2* M3 | [F]ACAGTATGTTTGCCTCAGCG | STS |
|  |  | [R] GTAAATCCACCCAGCCAGTC |  |
| *SSIIa* | *SSII-3* M1* | [F] CCAATACCGTAAACTAGCGACTATG | STS |
|  |  | [R] TACAGGTAGAATGGCAGTGGTG |  |
|  | *SSII-3* M2* | [F] GGTTCTCGGTGAAGATGGC | CAPS (*Ban II*) |
|  |  | [R] GTGGTCCCAGCTGAGGTCC |  |
|  | *SSII-3* M3 | [F] AACTGACTCATACACGGATAACG | CAPS (*Nhe I*) |
|  |  | [R] CACGCACGAACGGAAACC |  |
| *SSIIIb* | *SSIII-1* M1* | [F] AAGAAGGGAAGGGAGTCAGC | SSR |
|  |  | [R] GCCATCTCCATTGCCAGC |  |
|  | *SSIII-1* M2* | [F] CAAGCAATGATTCAGGCACA | CAPS (*EcoR I*) |
|  |  | [R] GGAGACAGGAGCAAAAGGC |  |
|  | *SSIII-1* M3* | [F] CAAATCAACTGTAAGTGCTGGAG | CAPS (*Nde I*) |
|  |  | [R] GAGAACGGAGAAAATGGCAT |  |
|  | *SSIII-1* t1 | [F] GGAGCAATAGGTGGTTCAA | CAPs(*Eco R*72I) |
|  |  | [R] GCCAAATCTACTCTCGTCA |  |
| *SSIIIa* | *SSIII-2* M1* | [F] AAGTCCTTCGGCTTACTATTCC | CAPS (*Xba I*) |
|  |  | [R] GGAGAAGGAACATAACAGGGAC |  |
|  | *SSIII-2* M2* | [F] GAACTTGTGCCTTAAGCTGACTG | STS |
|  |  | [R] GGAATAGTAAGCCGAAGGACTT |  |
| *SSIVa* | *SSIV-1* M1* | [F]CATTGTGTCTTGAAGTCTGTGCT | CAPS (*Nde I*) |
|  |  | [R] CGATGGGTTAGTGCTGTGG |  |
| *SSIVb* | *SSIV-2* M1* | [F] CTTCTGATTGATGGTTGGTTGC | CAPS (*Sph I*) |
|  |  | [R] GGAAGAATAATCTCTACTAGGTGGC |  |
|  | *SSIV-2* M2 | [F] TTCCCTTGGTGGTGCGTG | STS |
|  |  | [R] TAAAGCGTTCCGACAGTA |  |
|  | *SSIV-2* M3 | [F] TCAAGTATGGTTTACCTATG | CAPS (*Eco72 I*) |
|  |  | [R] TTTCCCAATGACTTCTAA |  |
| *SBE1* | *SBE1* M1* | [F] TGCTACATAACACGCATACAAAGT | STS |
|  |  | [R] AGACAAAAGCGAAAGGTAATGAG |  |
|  | *SBE1* M2 | [F] GTGGGGAAAACAAGTAAGTCTG | STS |
|  |  | [R] AGTTCCATCAGAAGAATCAGGG |  |
|  | *SBE1* M3 | [F] GGAAATGGGAGTCGCC | STS |
|  |  | [R] CGAAGAAACCACGCTCA |  |
| *BEIIb* | *SBE3* M1* | [F] AAGGTTAGCATTGGTTGGTGAG | STS |
|  |  | [R] TCTCCTTGAACAGCGACAGC |  |
|  | *SBE3* t1 | [F] TTCCATTATTTCTTTGCA | STS |
|  |  | [R]TATCCTCCCTGAACCAC |  |
| *BEIIa* | *SBE4* M1* | [F] CACCAATTATATTAGCGTGCTCC | STS |
|  |  | [R] CGTGGCTCTTGGCTCTCTTG |  |
|  | *SBE4* M2* | [F] CCATCACCTCAAATACATCACTC | STS |
|  |  | [R] AGACTGGAATGCCCCTTAGG |  |
| *ISA1* | *ISA* M1* | [F] ATAGATGCTAATGTGATGTGGC | STS |
|  |  | [R] TGGTATAGGCACAACCGTAGA |  |
|  | *ISA* M2 | [F] ACAAGCACACGACACCTA | CAPS (*Hind III*) |
|  |  | [R] CAACAAACCAAACTCATT |  |
|  | *ISA* M3 | [F] TGTGGGAATACCTTCAACTG | STS |
|  |  | [R] ATAAAACCCTTACAGGCTTG |  |
| *PUL* | *PUL* M1* | [F] AGAGAAGGAGAAAGAAGTGGAGAC | STS |
|  |  | [R] GTCCAAACTGAATCACTCAATCG |  |
|  | *PUL* M2* | [F] GACAACCGTCCGCTTTAGTTTC | STS |
|  |  | [R] GCATTTGAGAGGGTTTGGATTC |  |
|  | *PUL* M3* | [F] CTGTATGGACTGAGTAGTCGATGG | STS |
|  |  | [R] TGAGCCTCATCTGCCAGAGT |  |
|  | *PUL* M4 | [F] TACACCATCCTCACTACCA | STS |
|  |  | [R] GCAACATCTAAAACACCAA |  |
|  | *PUL* M5 | [F] ATTGGCATTTGTAAGTTTC | STS |
|  |  | [R] CAATCTTGGTTTTATCCTG |  |
|  | *PUL* M6 | [F] ATTTAACTGTATGGACTGAG | STS |
|  |  | [R] GATACCAACCAAACAAGA |  |

* Indicates the core markers, and # represents the enzymes used for CAPS.

**Table S3 |** The results of gel consistency and apparent amylose content for 63 glutinous rice.

| Number | GC±SD (cm) | AAC±SD (%) | Number | GC±SD (cm) | AAC±SD (%) | Number | GC±SD (cm) | AAC±SD (%) |
| --- | --- | --- | --- | --- | --- | --- | --- | --- |
| 1 | 10.13±0.12 | NA | 22 | 10.17±0.15 | 0.19±0.00 | 43 | 9.87±0.12 | NA |
| 2 | 10.73±0.12 | NA | 23 | 11.20±0.37 | NA | 44 | 9.97±0.23 | NA |
| 3 | 11.37±0.35 | NA | 24 | 10.07±0.06 | NA | 45 | 9.96±0.27 | NA |
| 4 | 10.07±0.40 | NA | 25 | 9.97±1.25 | 0.66±0.05 | 46 | 9.32±0.08 | NA |
| 5 | 10.70±0.10 | NA | 26 | 10.17±0.25 | 0.80±0.02 | 47 | 7.13±1.66 | 0.32±0.02 |
| 6 | 11.00±0.40 | NA | 27 | 10.13±0.06 | NA | 48 | 9.60±0.30 | NA |
| 7 | 10.97±0.21 | 0.26±0.02 | 28 | 10.30±0.53 | 0.66±0.04 | 49 | 9.90±0.64 | NA |
| 8 | 10.54±0.30 | NA | 29 | 10.73±0.71 | 1.88±0.07 | 50 | 9.40±0.39 | NA |
| 9 | 10.87±0.23 | 0.53 | 30 | 10.45±0.35 | NA | 51 | 8.98±0.34 | 0.32±0.03 |
| 10 | 11.83±0.43 | NA | 31 | 10.43±0.35 | NA | 52 | 9.92±0.40 | NA |
| 11 | 10.72±0.24 | NA | 32 | 9.88±0.39 | NA | 53 | 10.18±0.36 | NA |
| 12 | 10.00±0.45 | NA | 33 | 9.83±0.15 | NA | 54 | 10.03±0.23 | NA |
| 13 | 10.97±0.06 | NA | 34 | 10.50±0.32 | 0.26±0.01 | 55 | 8.50±0.10 | NA |
| 14 | 10.67±0.15 | NA | 35 | 10.55±0.29 | NA | 56 | 10.30±0.37 | NA |
| 15 | 10.00±0.36 | NA | 36 | 9.52±0.26 | NA | 57 | 9.52±0.55 | NA |
| 16 | 10.30±0.30 | NA | 37 | 10.28±0.41 | NA | 58 | 10.22±0.50 | NA |
| 17 | 11.27±0.46 | NA | 38 | 9.65±0.63 | NA | 59 | 10.07±0.18 | NA |
| 18 | 10.42±0.28 | 0.46±0.04 | 39 | 9.75±0.60 | 0.19±0.02 | 60 | 10.74±0.38 | NA |
| 19 | 9.33±0.51 | 0.19±0.01 | 40 | 10.72±0.54 | NA | 61 | 10.50±0.42 | NA |
| 20 | 10.50±0.40 | 0.19±0.00 | 41 | 10.98±0.34 | NA | 62 | 10.92±0.22 | NA |
| 21 | 10.20±0.43 | 0.32±0.02 | 42 | 11.35±0.30 | NA | 63 | 10.83±0.58 | NA |

GC: gel consistency; AAC: apparent amylose content; SD: the standard deviation.

NA indicates the AAC of glutinous rice accessions was too less to accurately measure by the Chinese national standards GB/T 15683-1995.

**Table S4 |** The results of RVA profile characteristics for 63 glutinous rices.

| Traits | Number of accessions | | | | | | | | | |
| --- | --- | --- | --- | --- | --- | --- | --- | --- | --- | --- |
|  | 1 | 2 | 3 | 4 | 5 | 6 | 7 | 8 | 9 | 10 |
| PKV (cp) | 2600.00±25.32 | 1989.00±0.00 | 2596.00±110.49 | 1872.00±9.90 | 2014.50±2.12 | 2442.00±22.58 | 2328.33±43.10 | 2283.00±7.07 | 2760.00±59.40 | 1654.50±20.51 |
| HPV (cp) | 1010.75±44.88 | 960.00±86.27 | 1012.25±17.46 | 776.00±4.24 | 734.00±4.24 | 984.75±28.92 | 1051.67±23.12 | 948.00±7.07 | 1051.50±13.44 | 590.00±36.77 |
| CPV (cp) | 1342.75±47.97 | 1269.50±126.57 | 1307.75±24.80 | 975.50±9.19 | 950.50±0.71 | 1351.50±25.27 | 1375.33±46.82 | 1225.50±4.95 | 1496.50±65.76 | 802.50±51.62 |
| BDV (cp) | 1589.25±50.28 | 1029.00±86.27 | 1583.75±95.53 | 1096.00±5.66 | 1280.50±6.36 | 1457.25±18.71 | 1276.67±46.61 | 1335.00±0.00 | 1708.50±72.83 | 1064.50±16.26 |
| SBV (cp) | -1257.25±45.63 | -719.50±126.57 | -1288.25±88.45 | -896.50±0.71 | -1064.00±2.83 | -1090.50±15.15 | -953.00±37.75 | -1057.50±2.12 | -1263.50±125.16 | -852.00±31.11 |
| CSV (cp) | 332.00±6.63 | 309.50±40.31 | 295.50±11.90 | 199.50±4.95 | 216.50±3.54 | 366.75±4.11 | 323.67±30.62 | 277.50±2.12 | 445.00±52.33 | 212.50±14.85 |
| PeT (min) | 3.78±0.06 | 3.87±0.09 | 3.50±0.04 | 3.77±0.05 | 3.47±0.00 | 3.80±0.00 | 3.89±0.04 | 3.60±0.00 | 3.53±0.00 | 3.47±0.00 |
| PaT (℃) | 72.76±0.43 | 73.33±1.10 | 71.14±0.38 | 72.95±0.64 | 69.28±0.04 | 73.00±0.52 | 73.17±0.53 | 68.95±0.64 | 70.95±0.07 | 70.88±0.04 |

Table S4 Continued

| Traits | Number of accessions | | | | | | | | | |
| --- | --- | --- | --- | --- | --- | --- | --- | --- | --- | --- |
|  | 11 | 12 | 13 | 14 | 15 | 16 | 17 | 18 | 19 | 20 |
| PKV (cp) | 2936.00±31.11 | 1442.33±87.13 | 2246.00±12.00 | 2693.00±8.49 | 2427.00±95.39 | 2244.00±76.37 | 1625.50±28.99 | 1440.00±138.16 | 2355.33±23.46 | 2780.67±65.32 |
| HPV (cp) | 887.00±9.90 | 535.67±78.93 | 970.33±0.58 | 1013.50±7.78 | 1178.67±125.61 | 1042.50±6.36 | 786.00±22.63 | 515.67±20.84 | 908.33±63.79 | 1285.33±15.04 |
| CPV (cp) | 1251.50±30.41 | 691.33±94.16 | 1233.67±28.36 | 1406.00±29.70 | 1544.67±147.82 | 1378.50±28.99 | 1008.00±29.70 | 686.33±25.66 | 1142.00±62.45 | 1727.33±21.20 |
| BDV (cp) | 2049.00±21.21 | 906.67±8.50 | 1275.67±12.01 | 1679.50±16.26 | 1248.33±30.29 | 1201.50±82.73 | 839.50±6.36 | 924.33±117.64 | 1447.00±46.87 | 1495.33±50.52 |
| SBV (cp) | -1684.50±0.71 | -751.00±10.44 | -1012.33±28.99 | -1287.00±38.18 | -882.33±52.62 | -865.50±47.38 | -617.50±0.71 | -753.67±112.72 | -1213.33±43.02 | -1053.33±44.28 |
| CSV (cp) | 364.50±20.51 | 155.67±16.04 | 263.33±27.79 | 392.50±21.92 | 366.00±23.58 | 336.00±35.36 | 222.00±7.07 | 170.67±4.93 | 233.67±9.45 | 442.00±6.24 |
| PeT (min) | 3.47±0.00 | 3.58±0.04 | 3.56±0.04 | 3.57±0.05 | 3.91±0.10 | 3.80±0.00 | 3.87±0.00 | 3.73±0.18 | 3.78±0.04 | 3.98±0.04 |
| PaT (℃) | 69.70±0.57 | 71.25±0.56 | 70.17±0.06 | 70.93±0.11 | 73.20±0.52 | 72.58±0.11 | 73.43±0.04 | 73.18±1.76 | 72.57±0.83 | 73.67±0.46 |

Table S4 Continued

| Traits | Number of accessions | | | | | | | | | |
| --- | --- | --- | --- | --- | --- | --- | --- | --- | --- | --- |
|  | 21 | 22 | 23 | 24 | 25 | 26 | 27 | 28 | 29 | 30 |
| PKV (cp) | 2406.50±21.02 | 1493.50±188.80 | 1215.00±457.16 | 1327.00±101.82 | 2171.33±106.97 | 2214.50±133.64 | 2062.00±123.04 | 2815.00±435.58 | 2410.00±29.70 | 2269.67±10.60 |
| HPV (cp) | 1052.50±80.10 | 358.50±64.35 | 301.00±133.19 | 410.00±43.84 | 1217.00±36.72 | 959.50±10.61 | 724.00±21.21 | 1217.00±98.99 | 1091.50±91.22 | 805.33±29.02 |
| CPV (cp) | 1359.75±105.79 | 502.00±90.51 | 427.67±165.98 | 564.00±56.57 | 1618.00±48.87 | 1280.00±5.66 | 982.50±26.16 | 1628.00±168.29 | 1453.50±144.96 | 1075.67±31.66 |
| BDV (cp) | 1354.00±73.39 | 1135.00±124.45 | 914.00±331.48 | 917.00±57.98 | 954.33±70.61 | 1255.00±123.04 | 1338.00±101.82 | 1598.00±336.58 | 1318.50±61.52 | 1464.33±35.92 |
| SBV (cp) | -1046.75±97.05 | -991.50±98.29 | -787.33±302.70 | -763.00±45.25 | -553.33±58.82 | -934.50±127.99 | -1079.50±96.87 | -1187.00±267.29 | -956.50±115.26 | -1194.00±37.80 |
| CSV (cp) | 307.25±31.93 | 143.50±26.16 | 126.67±32.88 | 154.00±12.73 | 401.00±12.17 | 320.50±4.95 | 258.50±4.95 | 411.00±69.30 | 362.00±53.74 | 270.33±3.79 |
| PeT (min) | 3.85±0.06 | 3.50±0.05 | 3.51±0.14 | 3.63±0.05 | 4.58±0.04 | 3.97±0.05 | 3.47±0.00 | 4.27±0.00 | 4.30±0.05 | 3.47±0.00 |
| PaT (℃) | 72.61±1.20 | 70.93±0.04 | 71.55±0.40 | 72.58±0.04 | 80.73±0.03 | 73.45±1.20 | 70.55±0.64 | 81.53±0.04 | 82.05±0.64 | 70.35±0.44 |

Table S4 Continued

| Traits | Number of accessions | | | | | | | | | |
| --- | --- | --- | --- | --- | --- | --- | --- | --- | --- | --- |
|  | 31 | 32 | 33 | 34 | 35 | 36 | 37 | 38 | 39 | 40 |
| PKV (cp) | 2606.00±173.23 | 2621.67±50.08 | 2540.00±32.53 | 2359.67±690.06 | 2308.67±46.00 | 2667.33±195.40 | 2516.67±136.08 | 2421.50±75.66 | 2276.50±81.32 | 2195.33±52.00 |
| HPV (cp) | 1009.00±199.55 | 1126.33±63.79 | 1349.00±38.18 | 1061.67±532.53 | 1068.00±10.54 | 1401.00±65.51 | 1400.67±94.09 | 1312.50±28.99 | 1280.00±96.17 | 990.67±44.84 |
| CPV (cp) | 1353.33±292.03 | 1510.00±83.74 | 1820.00±60.81 | 1437.33±705.86 | 1391.33±10.41 | 1817.33±110.55 | 1870.67±114.18 | 1739.00±25.46 | 1709.00±98.99 | 1254.00±53.56 |
| BDV (cp) | 1597.00±82.40 | 1495.33±107.54 | 1191.00±5.66 | 1298.00±213.13 | 1240.67±52.81 | 1266.33±130.48 | 1116.00±60.65 | 1109.00±46.67 | 996.50±14.85 | 1204.67±14.50 |
| SBV (cp) | -1252.67±126.43 | -1111.67±126.22 | -720.00±28.28 | -922.33±145.23 | -917.33±36.07 | -850.00±84.86 | -646.00±42.23 | -682.50±50.20 | -567.50±17.68 | -941.33±10.02 |
| CSV (cp) | 344.33±107.87 | 383.67±20.03 | 471.00±22.63 | 375.67±174.32 | 323.33±19.73 | 416.33±46.05 | 470.00±22.54 | 426.50±3.54 | 429.00±2.83 | 263.33±9.45 |
| PeT (min) | 3.49±0.08 | 3.69±0.08 | 4.40±0.00 | 3.98±0.62 | 3.98±0.04 | 4.42±0.04 | 4.51±0.04 | 4.40±0.00 | 4.47±0.09 | 3.84±0.04 |
| PaT (℃) | 69.82±0.41 | 71.75±0.05 | 80.70±0.10 | 77.52±6.35 | 74.78±0.51 | 81.78±0.49 | 81.78±0.49 | 80.80±0.00 | 80.30±0.42 | 72.85±0.48 |

Table S4 Continued

| Traits | Number of accessions | | | | | | | | | |
| --- | --- | --- | --- | --- | --- | --- | --- | --- | --- | --- |
|  | 41 | 42 | 43 | 44 | 45 | 46 | 47 | 48 | 49 | 50 |
| PKV (cp) | 2108.00±31.11 | 2639.00±112.09 | 2220.00±514.57 | 2482.00±342.24 | 3524.00±7.07 | 2551.50±147.79 | 2747.50±132.23 | 2055.33±427.10 | 1349.00±295.57 | 2632.50±23.33 |
| HPV (cp) | 934.00±12.73 | 924.67±19.22 | 934.00±194.24 | 1031.00±130.11 | 1853.50±103.94 | 1036.00±100.41 | 1362.00±32.53 | 837.67±236.79 | 401.00±67.88 | 1098.50±13.44 |
| CPV (cp) | 1177.00±4.24 | 1204.67±34.67 | 1243.00±301.29 | 1399.00±185.26 | 3165.00±67.88 | 1394.00±117.38 | 1818.50±45.96 | 1099.33±320.15 | 557.00±97.58 | 1408.50±3.54 |
| BDV (cp) | 1174.00±18.38 | 1714.33±93.84 | 1286.00±320.66 | 1451.00±212.13 | 1670.50±96.87 | 1515.50±47.38 | 1385.50±99.70 | 1217.67±235.28 | 948.00±227.69 | 1534.00±9.90 |
| SBV (cp) | -931.00±26.87 | -1434.33±81.29 | -977.00±213.85 | -1083.00±156.98 | -359.00±74.95 | -1157.50±30.41 | -929.00±86.27 | -956.00±187.93 | -792.00±197.99 | -1224.00±19.80 |
| CSV (cp) | 243.00±8.49 | 280.00±15.72 | 309.00±107.06 | 368.00±55.15 | 1311.50±171.83 | 358.00±16.97 | 456.50±13.44 | 261.67±83.50 | 156.00±29.70 | 310.00±9.90 |
| PeT (min) | 3.93±0.00 | 3.69±0.04 | 3.82±0.04 | 3.80±0.00 | 3.80±0.00 | 3.83±0.05 | 4.43±0.05 | 3.84±0.08 | 3.70±0.05 | 3.80±0.00 |
| PaT (℃) | 73.43±0.04 | 72.27±0.45 | 72.88±0.49 | 73.88±0.60 | 74.23±0.18 | 72.55±0.00 | 82.33±0.11 | 74.28±0.08 | 73.90±0.57 | 73.03±0.60 |

| Traits | Number of accessions | | | | | | | | | |
| --- | --- | --- | --- | --- | --- | --- | --- | --- | --- | --- |
|  | 51 | 52 | 53 | 54 | 55 | 56 | 57 | 58 | 59 | 60 |
| PKV (cp) | 1190.50±36.06 | 2728.00±246.07 | 2361.33±199.02 | 2639.33±73.65 | 2733.00±94.98 | 2065.00±29.70 | 3183.67±568.92 | 2542.00±117.38 | 2443.33±180.91 | 1825.50±169.00 |
| HPV (cp) | 331.00±87.68 | 1331.50±139.30 | 1023.67±73.28 | 1232.67±40.38 | 1295.00±27.62 | 808.00±31.11 | 1126.67±219.86 | 919.50±57.28 | 994.67±95.62 | 549.00±49.50 |
| CPV (cp) | 465.50±113.84 | 1791.50±156.27 | 1362.33±84.01 | 1643.00±41.58 | 1717.33±35.22 | 1047.50±31.82 | 1519.67±331.16 | 1185.00±72.12 | 1349.00±145.75 | 739.50±60.10 |
| BDV (cp) | 859.50±51.62 | 1396.50±106.77 | 1337.67±134.70 | 1406.67±111.47 | 1438.00±108.68 | 1257.00±60.81 | 2057.00±352.68 | 1622.50±60.10 | 1448.67±94.32 | 1276.50±119.50 |
| SBV (cp) | -725.00±77.78 | -936.50±89.80 | -999.00±115.33 | -996.33±113.74 | -1015.67±121.99 | -1017.50±61.52 | -1664.00±250.09 | -1357.00±45.25 | -1094.33±45.54 | -1086.00±108.89 |
| CSV (cp) | 134.50±26.16 | 460.00±16.97 | 338.67±28.38 | 410.33±4.62 | 422.33±13.65 | 239.50±0.71 | 393.00±111.73 | 265.50±14.85 | 354.33±52.39 | 190.50±10.61 |
| PeT (min) | 3.67±0.09 | 4.33±0.00 | 3.69±0.08 | 4.24±0.04 | 4.29±0.08 | 3.57±0.05 | 3.58±0.04 | 3.57±0.05 | 3.87±0.00 | 3.37±0.05 |
| PaT (℃) | 74.20±0.00 | 80.75±0.07 | 72.35±0.48 | 80.43±0.43 | 80.72±0.03 | 70.90±0.00 | 70.63±0.51 | 70.88±0.04 | 72.60±0.05 | 70.48±0.53 |

Table S4 Continued

Table S4 Continued

| Traits | Number of accessions | | |
| --- | --- | --- | --- |
|  | 61 | 62 | 63 |
| PKV (cp) | 1615.00±65.05 | 1706.00±14.14 | 1484.50±27.58 |
| HPV (cp) | 568.50±10.61 | 567.50±19.09 | 509.00±5.66 |
| CPV (cp) | 758.50±10.61 | 753.00±16.97 | 685.00±2.83 |
| BDV (cp) | 1046.50±54.45 | 1138.50±4.95 | 975.50±21.92 |
| SBV (cp) | -856.50±54.45 | -953.00±2.83 | -799.50±24.75 |
| CSV (cp) | 190.00±0.00 | 185.50±2.12 | 176.00±2.83 |
| PeT (min) | 3.43±0.05 | 3.40±0.00 | 3.43±0.05 |
| PaT (℃) | 70.55±0.64 | 70.53±0.39 | 70.53±0.60 |

PKV: peak viscosity; HPV: hot paste viscosity; CPV: cool paste viscosity; BDV: breakdown viscosity; SBV: setback viscosity; CSV: Consistence viscosity; PeT: peak time; PaT: pasting temperature; SD: the standard deviation.

**Table S5 |** The results of thermal and retrogradation properties for 63 glutinous rice.

| Number | To±SD (℃) | Tp±SD (℃) | Tc±SD (℃) | △T1/2±SD (℃) | △Hg±SD (J/g) | △Hr±SD (J/g) | R%±SD |
| --- | --- | --- | --- | --- | --- | --- | --- |
| 1 | 65.32±0.24 | 71.99±0.19 | 80.87±0.44 | 9.26±0.34 | 11.36±0.44 | 2.90±0.13 | 0.26±0.01 |
| 2 | 66.65±0.36 | 72.92±0.11 | 81.20±0.67 | 8.82±0.58 | 10.58±1.01 | 3.02±0.32 | 0.30±0.01 |
| 3 | 64.38±0.06 | 70.62±0.19 | 77.42±0.50 | 7.54±0.25 | 10.29±0.26 | 2.01±0.16 | 0.20±0.01 |
| 4 | 66.12±0.24 | 73.67±0.37 | 81.41±0.33 | 9.05±0.01 | 10.66±0.21 | 4.30±0.27 | 0.40±0.02 |
| 5 | 64.56±0.13 | 70.23±0.01 | 77.98±0.07 | 7.64±0.01 | 10.09±0.34 | 2.34±0.21 | 0.23±0.01 |
| 6 | 64.62±0.24 | 72.31±0.11 | 80.03±0.29 | 8.88±0.26 | 11.02±0.29 | 3.07±0.33 | 0.28±0.02 |
| 7 | 65.32±0.30 | 72.72±0.46 | 82.20±0.47 | 10.11±0.06 | 11.22±0.08 | 3.36±0.21 | 0.30±0.02 |
| 8 | 62.02±0.12 | 68.97±0.11 | 79.08±0.03 | 9.86±0.01 | 10.15±0.16 | 2.45±0.02 | 0.24±0.00 |
| 9 | 64.46±0.91 | 70.23±0.88 | 78.17±0.33 | 7.89±0.28 | 10.25±1.43 | 2.82±0.53 | 0.28±0.01 |
| 10 | 65.05±0.26 | 70.87±0.01 | 79.70±0.03 | 8.51±0.11 | 10.21±1.27 | 3.87±0.81 | 0.39±0.13 |
| 11 | 63.40±0.02 | 69.31±0.13 | 76.99±1.19 | 8.14±0.70 | 10.41±1.03 | 2.75±0.21 | 0.26±0.01 |
| 12 | 64.47±0.12 | 70.67±0.12 | 79.64±0.30 | 9.08±0.23 | 10.80±0.54 | 2.94±0.08 | 0.27±0.01 |
| 13 | 63.85±0.13 | 69.83±0.14 | 78.61±0.19 | 8.62±0.01 | 9.72±0.73 | 2.04±1.11 | 0.21±0.13 |
| 14 | 63.68±0.10 | 70.25±0.25 | 78.29±0.28 | 9.20±0.18 | 11.16±0.18 | 3.05±0.32 | 0.27±0.02 |
| 15 | 65.44±0.57 | 72.90±0.51 | 80.50±0.74 | 8.56±0.15 | 10.92±0.52 | 2.37±0.42 | 0.22±0.05 |
| 16 | 65.93±0.96 | 73.29±0.12 | 79.92±0.24 | 8.35±0.30 | 11.12±0.36 | 2.51±0.90 | 0.22±0.07 |
| 17 | 65.84±0.09 | 72.83±0.11 | 81.15±0.45 | 8.83±0.24 | 10.82±0.35 | 2.13±0.81 | 0.20±0.07 |
| 18 | 67.25±0.37 | 74.21±0.24 | 82.72±0.21 | 8.93±0.04 | 10.80±0.13 | 3.05±0.08 | 0.28±0.00 |
| 19 | 67.33±0.21 | 72.96±0.62 | 80.44±0.38 | 7.54±0.05 | 9.68±1.99 | 2.66±0.45 | 0.29±0.11 |
| 20 | 67.40±0.49 | 73.09±0.74 | 82.07±1.17 | 8.78±0.42 | 11.77±0.37 | 3.46±0.06 | 0.29±0.00 |
| 21 | 66.98±0.06 | 73.03±0.33 | 81.94±0.89 | 8.57±0.49 | 10.72±0.52 | 3.07±0.11 | 0.29±0.00 |
| 22 | 64.33±0.88 | 70.34±0.51 | 79.09±0.35 | 8.36±0.69 | 9.77±1.36 | 1.71±0.64 | 0.17±0.04 |
| 23 | 64.29±0.12 | 70.89±0.13 | 79.11±0.36 | 8.42±0.17 | 9.51±1.01 | 1.11±0.24 | 0.12±0.01 |
| 24 | 65.68±0.17 | 71.74±0.22 | 79.30±0.34 | 8.00±0.19 | 11.30±0.25 | 1.72±0.02 | 0.15±0.00 |
| 25 | 74.90±0.08 | 80.61±0.10 | 87.37±0.19 | 7.21±0.05 | 11.89±0.14 | 5.98±0.49 | 0.50±0.05 |
| 26 | 65.76±0.45 | 72.56±0.47 | 82.09±0.10 | 9.59±0.07 | 11.28±0.23 | 2.59±0.00 | 0.23±0.00 |
| 27 | 64.67±0.02 | 70.31±0.11 | 77.64±0.28 | 7.52±0.17 | 10.90±0.33 | 2.11±0.13 | 0.19±0.01 |
| 28 | 76.31±0.56 | 80.53±0.45 | 85.98±0.59 | 5.55±0.01 | 12.78±0.33 | 6.17±0.15 | 0.48±0.00 |
| 29 | 77.09±0.52 | 81.21±0.48 | 86.60±0.37 | 5.50±0.06 | 13.26±0.53 | 6.11±0.23 | 0.46±0.04 |
| 30 | 64.29±0.06 | 70.05±0.25 | 77.04±0.59 | 7.88±0.47 | 10.03±0.08 | 2.16±0.51 | 0.22±0.05 |
| 31 | 63.96±0.15 | 69.95±0.18 | 77.82±0.30 | 8.09±0.26 | 8.66±0.06 | 3.38±1.12 | 0.39±0.13 |
| 32 | 65.20±0.10 | 70.83±0.10 | 78.56±0.34 | 7.73±0.03 | 9.97±0.54 | 2.81±0.01 | 0.28±0.02 |
| 33 | 75.64±0.33 | 80.47±0.36 | 86.33±0.15 | 6.27±0.10 | 11.69±0.52 | 6.03±0.17 | 0.52±0.01 |
| 34 | 76.00±0.19 | 80.61±0.36 | 86.31±0.56 | 5.96±0.21 | 12.38±0.42 | 6.56±1.51 | 0.53±0.14 |
| 35 | 68.65±0.07 | 74.47±0.35 | 82.07±0.92 | 7.87±0.45 | 9.38±1.37 | 2.66±0.64 | 0.28±0.03 |
| 36 | 77.30±0.57 | 81.73±0.53 | 87.35±0.58 | 5.85±0.01 | 11.11±2.09 | 6.72±0.57 | 0.62±0.17 |
| 37 | 77.26±0.20 | 81.68±0.24 | 87.18±0.01 | 5.79±0.15 | 10.65±0.32 | 6.27±0.16 | 0.59±0.00 |
| 38 | 74.99±0.38 | 80.12±0.36 | 86.23±0.03 | 6.52±0.20 | 12.10±0.70 | 5.59±0.59 | 0.46±0.02 |
| 39 | 75.52±0.12 | 80.51±0.28 | 86.95±0.15 | 6.62±0.03 | 10.94±0.14 | 5.54±0.21 | 0.51±0.03 |
| 40 | 65.59±0.56 | 72.34±0.30 | 80.71±0.04 | 8.95±0.32 | 10.08±0.37 | 2.60±0.43 | 0.26±0.05 |
| 41 | 67.23±0.04 | 72.30±0.36 | 82.89±0.05 | 9.00±0.03 | 10.03±0.64 | 2.36±0.25 | 0.24±0.01 |
| 42 | 65.78±0.30 | 71.44±0.23 | 78.64±0.69 | 7.50±0.40 | 9.95±0.25 | 2.50±0.28 | 0.25±0.02 |
| 43 | 66.99±0.69 | 72.72±0.73 | 82.49±0.54 | 8.80±0.08 | 10.58±0.33 | 2.99±0.52 | 0.28±0.06 |
| 44 | 67.63±0.37 | 73.24±0.24 | 80.29±0.40 | 7.38±0.01 | 10.05±0.17 | 3.01±0.13 | 0.30±0.01 |
| 45 | 66.57±0.07 | 71.02±0.06 | 87.16±0.12 | 5.59±0.09 | 7.70±0.05 | 0.70±0.13 | 0.09±0.02 |
| 46 | 65.50±0.34 | 72.41±0.27 | 80.34±0.44 | 8.81±0.41 | 9.62±1.16 | 2.40±0.31 | 0.25±0.02 |
| 47 | 77.57±0.39 | 82.12±0.51 | 87.16±0.59 | 5.73±0.22 | 11.70±1.86 | 5.95±0.12 | 0.57±0.11 |
| 48 | 68.12±0.03 | 73.50±0.11 | 80.67±0.78 | 7.25±0.46 | 9.20±0.74 | 2.06±0.61 | 0.22±0.05 |
| 49 | 67.15±0.04 | 73.44±0.11 | 80.08±0.11 | 7.54±0.20 | 8.35±0.29 | 0.95±0.12 | 0.11±0.00 |
| 50 | 66.92±0.86 | 73.15±0.00 | 80.57±0.33 | 7.62±0.76 | 10.72±0.74 | 3.04±0.64 | 0.26±0.04 |
| 51 | 67.32±0.86 | 73.26±0.35 | 80.52±0.01 | 7.82±0.30 | 10.97±0.74 | 2.90±0.39 | 0.26±0.05 |
| 52 | 75.24±0.09 | 80.31±0.42 | 86.09±0.08 | 6.27±0.18 | 12.14±0.38 | 6.54±1.41 | 0.54±0.10 |
| 53 | 65.18±0.24 | 71.16±0.12 | 79.27±0.01 | 8.29±0.23 | 10.17±0.71 | 2.33±0.10 | 0.23±0.01 |
| 54 | 75.49±0.08 | 80.10±0.17 | 85.35±0.42 | 5.63±0.24 | 11.80±0.68 | 5.87±0.12 | 0.50±0.02 |
| 55 | 75.49±0.16 | 80.36±0.01 | 85.99±0.11 | 6.10±0.11 | 12.19±0.01 | 6.99±0.14 | 0.57±0.01 |
| 56 | 64.89±0.19 | 70.72±0.01 | 78.43±0.13 | 7.97±0.22 | 9.37±0.35 | 1.70±0.08 | 0.18±0.00 |
| 57 | 63.37±0.26 | 69.64±0.60 | 79.44±0.25 | 9.36±0.29 | 11.45±0.32 | 3.45±0.59 | 0.30±0.04 |
| 58 | 64.34±1.52 | 70.51±0.98 | 77.81±1.10 | 8.24±0.13 | 9.62±0.82 | 2.35±0.13 | 0.25±0.03 |
| 59 | 65.09±0.08 | 72.49±0.49 | 81.50±0.20 | 9.89±0.18 | 10.38±0.11 | 3.25±0.54 | 0.31±0.06 |
| 60 | 64.27±0.28 | 69.78±0.47 | 78.26±0.70 | 7.79±0.17 | 9.48±0.64 | 1.88±0.01 | 0.20±0.01 |
| 61 | 63.80±0.17 | 69.92±0.12 | 79.11±0.24 | 9.06±0.17 | 10.28±0.62 | 1.96±0.71 | 0.19±0.06 |
| 62 | 64.29±0.15 | 69.70±0.06 | 78.26±0.46 | 8.07±0.10 | 10.04±0.08 | 2.69±0.01 | 0.27±0.00 |
| 63 | 63.41±0.22 | 70.10±0.11 | 79.16±0.18 | 9.40±0.25 | 9.72±0.04 | 2.56±0.23 | 0.26±0.02 |

To: onset temperature; Tp: peak temperature; Tc: conclusion temperature; ∆T1/2: width at half peak height; ∆Hg: enthalpy of gelatinization; ∆Hr: enthalpy of retrogradation; R%: percentage of retrogradation; SD: the standard deviation.

**Table S6 |** The Q-values of 63 glutinous accessions.

| Number | Inferred | clusters | Number | Inferred | clusters |
| --- | --- | --- | --- | --- | --- |
| 1 | 0.003 | 0.997 | 33 | 0.999 | 0.001 |
| 2 | 0.998 | 0.002 | 34 | 0.999 | 0.001 |
| 3 | 0.001 | 0.999 | 35 | 0.999 | 0.001 |
| 4 | 0.002 | 0.998 | 36 | 0.004 | 0.996 |
| 5 | 0.999 | 0.001 | 37 | 0.009 | 0.991 |
| 6 | 0.001 | 0.999 | 38 | 0.002 | 0.998 |
| 7 | 0.999 | 0.001 | 39 | 0.004 | 0.996 |
| 8 | 0.788 | 0.212 | 40 | 0.998 | 0.002 |
| 9 | 0.012 | 0.988 | 41 | 0.998 | 0.002 |
| 10 | 0.998 | 0.002 | 42 | 0.001 | 0.999 |
| 11 | 0.124 | 0.876 | 43 | 0.001 | 0.999 |
| 12 | 0.882 | 0.118 | 44 | 0.064 | 0.936 |
| 13 | 0.006 | 0.994 | 45 | 0.001 | 0.999 |
| 14 | 0.003 | 0.997 | 46 | 0.001 | 0.999 |
| 15 | 0.001 | 0.999 | 47 | 0.001 | 0.999 |
| 16 | 0.002 | 0.998 | 48 | 0.054 | 0.946 |
| 17 | 0.999 | 0.001 | 49 | 0.002 | 0.998 |
| 18 | 0.792 | 0.208 | 50 | 0.035 | 0.965 |
| 19 | 0.003 | 0.997 | 51 | 0.046 | 0.954 |
| 20 | 0.864 | 0.136 | 52 | 0.005 | 0.995 |
| 21 | 0.999 | 0.001 | 53 | 0.001 | 0.999 |
| 22 | 0.002 | 0.998 | 54 | 0.145 | 0.855 |
| 23 | 0.002 | 0.998 | 55 | 0.001 | 0.999 |
| 24 | 0.003 | 0.997 | 56 | 0.001 | 0.999 |
| 25 | 0.002 | 0.998 | 57 | 0.001 | 0.999 |
| 26 | 0.669 | 0.331 | 58 | 0.001 | 0.999 |
| 27 | 0.001 | 0.999 | 59 | 0.001 | 0.999 |
| 28 | 0.16 | 0.84 | 60 | 0.999 | 0.001 |
| 29 | 0.999 | 0.001 | 61 | 0.999 | 0.001 |
| 30 | 0.001 | 0.999 | 62 | 0.972 | 0.028 |
| 31 | 0.001 | 0.999 | 63 | 0.999 | 0.001 |
| 32 | 0.041 | 0.959 |  |  |  |

**Table** **S7 |** The results of gene genotyping for 63 glutinous accessions.

|  | *AGPlar* M1 | *AGPlar* M2 | *AGPiso* M2 | *AGPiso* M3 | *AGPsma* M1 | *AGPsma* M2 | *GBSSII* | *SSI* M1 | *SSI* M2 | *SSI* M3 | *SSIIa* M1 | *SSIIa* M2 | *SSIIa* M3 | *SSIIb* M1 | *SSIIb* M2 | *SSIIb* M3 | *SSIIc* M1 | *SSIIc* M2 | *SSIIc* M3 | *SSIIc* M4 | *SSIIIa* M1 | *SSIIIa* M2 | *SSIIIb* M1 | *SSIIIb* M2 | *SSIIIb* M3 | *SSIIIb* t1 | *SSIVa* M1 | *SSIVb* M1 | *SSIVb* M2 | *SSIVb* M3 | *SBE1* M1 | *SBE1* M2 | *SBE1* M3 | *BEIIa* M1 | *BEIIa* M2 | *BEIIb* M1 | *BEIIb* t1 | *ISA1* M1 | *ISA1* M2 | *ISA1* M3 | *PUL* M1 | *PUL* M2 | *PUL* M3 | *PUL* M4 | *PUL* M5 | *PUL* M6 |
| --- | --- | --- | --- | --- | --- | --- | --- | --- | --- | --- | --- | --- | --- | --- | --- | --- | --- | --- | --- | --- | --- | --- | --- | --- | --- | --- | --- | --- | --- | --- | --- | --- | --- | --- | --- | --- | --- | --- | --- | --- | --- | --- | --- | --- | --- | --- |
| 1 | b | b | b | a | b | a | b | b | b | b | a | a | b | a | b | b | b | b | b | b | b | a | d | a | a | b | b | a | a | a | b | a | b | a | b | a | b | b | a | a | b | c | a | b | a | a |
| 2 | a | a | b | a | a | b | a | b | a | a | a | a | b | b | b | a | b | a | a | a | a | a | c | a | b | b | a | b | a | b | a | b | b | b | a | a | b | a | b | b | b | b | a | b | a | a |
| 3 | c | b | b | a | b | a | a | a | a | a | a | a | b | a | b | b | b | b | b | b | a | a | b | a | b | b | b | b | a | b | b | a | b | a | b | b | a | b | a | a | b | a | b | a | b | b |
| 4 | b | b | b | a | b | a | b | a | b | b | a | a | b | a | b | b | b | b | b | b | a | a | d | a | a | b | b | a | a | a | b | a | b | a | b | b | a | b | a | a | b | c | a | b | a | a |
| 5 | a | a | a | b | a | a | a | a | a | a | a | a | b | b | b | a | a | a | b | b | b | b | c | a | b | b | a | b | a | b | a | b | a | b | a | a | b | a | b | b | a | b | a | b | a | a |
| 6 | b | b | b | a | c | a | a | a | a | a | a | a | b | a | b | b | b | b | b | b | a | a | b | a | b | b | b | a | a | a | b | a | b | a | b | b | a | b | a | a | b | b | a | b | a | a |
| 7 | a | a | b | a | a | b | a | b | a | a | a | a | b | b | b | a | b | a | a | a | b | b | a | b | b | a | a | b | b | b | a | b | a | b | a | a | b | a | b | b | a | a | a | b | a | a |
| 8 | a | a | b | a | a | a | a | a | a | a | a | a | b | b | b | a | a | a | b | b | a | a | a | b | b | a | a | b | a | b | b | a | b | b | a | a | b | a | b | b | b | b | b | a | b | b |
| 9 | c | b | b | a | b | a | b | b | b | b | a | a | b | a | b | b | b | b | b | b | b | a | b | a | b | b | b | a | a | a | b | a | b | a | b | b | a | b | a | a | b | c | a | b | a | a |
| 10 | a | a | a | b | a | a | a | b | a | a | a | a | b | b | b | a | b | a | a | a | b | a | c | a | b | b | b | b | a | b | a | b | b | b | a | a | b | a | b | b | a | b | a | b | a | a |
| 11 | b | b | b | a | b | a | a | a | b | b | a | a | b | a | b | b | b | b | b | b | b | a | d | a | a | b | b | a | a | a | b | a | b | a | b | b | a | b | a | a | b | a | b | a | b | b |
| 12 | a | a | a | b | a | a | b | b | a | a | a | a | b | b | b | a | b | b | b | b | b | a | c | a | b | b | a | b | a | b | b | a | b | b | a | b | a | a | b | b | a | b | a | b | a | a |
| 13 | c | b | b | a | b | a | b | a | a | a | a | a | b | a | b | b | b | b | b | b | b | a | b | a | b | b | b | b | a | b | b | a | b | a | b | b | a | b | a | a | b | a | b | a | b | b |
| 14 | c | b | b | a | b | a | b | a | a | a | a | a | b | a | a | b | b | b | b | b | b | a | b | b | b | b | b | b | a | b | b | a | b | a | b | a | b | b | a | a | b | a | b | a | b | b |
| 15 | c | b | b | a | b | a | b | a | a | a | a | a | b | a | b | b | b | b | b | b | b | a | d | a | a | b | b | a | a | a | b | a | b | a | b | b | a | b | a | a | b | c | a | b | a | a |
| 16 | b | b | b | a | b | a | a | b | a | a | a | a | b | a | a | b | b | b | b | b | b | a | b | b | b | b | a | b | a | b | b | a | b | b | a | a | b | b | a | a | b | a | b | a | b | b |
| 17 | a | a | b | a | a | a | b | b | a | a | a | a | b | b | b | a | b | a | a | a | a | a | c | a | b | b | a | b | a | b | a | b | b | b | a | a | b | a | b | b | b | b | b | b | b | b |
| 18 | a | a | b | a | a | b | b | b | a | a | a | a | b | b | b | a | b | b | b | b | b | b | a | b | b | a | a | b | b | b | a | b | a | b | a | a | b | b | a | a | a | b | a | b | a | a |
| 19 | c | b | b | a | b | a | a | a | b | b | a | a | b | a | b | b | b | b | b | b | b | a | d | b | a | b | b | a | a | a | b | a | b | a | b | b | a | b | a | a | b | a | b | a | b | b |
| 20 | a | a | a | b | b | a | b | b | a | a | a | a | b | b | b | a | b | a | a | a | a | a | b | a | b | b | b | b | a | b | a | b | b | b | a | a | b | b | a | a | b | c | a | b | a | a |
| 21 | a | a | a | b | a | b | b | b | a | a | a | a | b | b | b | a | b | a | a | a | b | b | b | a | b | b | a | b | a | b | a | b | b | b | a | a | b | a | b | b | a | b | a | b | a | a |
| 22 | c | b | b | a | b | a | a | a | a | a | a | a | b | a | b | b | b | b | b | b | b | a | d | b | a | b | b | a | a | a | b | a | b | a | b | b | a | b | a | a | b | a | b | a | b | b |
| 23 | c | b | b | a | b | a | b | a | a | a | a | a | b | a | b | b | b | b | b | b | b | a | d | b | a | b | b | a | a | a | b | a | b | a | b | b | a | b | a | a | b | a | b | a | b | b |
| 24 | c | b | b | a | b | a | b | a | b | b | a | a | b | a | b | b | b | b | b | b | b | a | d | b | a | b | b | a | a | a | b | a | b | a | b | b | a | b | a | a | b | a | b | a | b | b |
| 25 | c | b | b | a | c | a | a | a | b | b | a | a | b | a | b | b | b | b | b | b | a | a | b | a | b | b | b | a | a | a | b | a | b | a | b | b | a | b | a | a | b | c | a | b | a | a |

Supplementary Table 7**|** Continued

|  | *AGPlar* M1 | *AGPlar* M2 | *AGPiso* M2 | *AGPiso* M3 | *AGPsma* M1 | *AGPsma* M2 | *GBSSII* | *SSI* M1 | *SSI* M2 | *SSI* M3 | *SSIIa* M1 | *SSIIa* M2 | *SSIIa* M3 | *SSIIb* M1 | *SSIIb* M2 | *SSIIb* M3 | *SSIIc* M1 | *SSIIc* M2 | *SSIIc* M3 | *SSIIc* M4 | *SSIIIa* M1 | *SSIIIa* M2 | *SSIIIb* M1 | *SSIIIb* M2 | *SSIIIb* M3 | *SSIIIb* t1 | *SSIVa* M1 | *SSIVb* M1 | *SSIVb* M2 | *SSIVb* M3 | *SBE1* M1 | *SBE1* M2 | *SBE1* M3 | *BEIIa* M1 | *BEIIa* M2 | *BEIIb* M1 | *BEIIb* t1 | *ISA1* M1 | *ISA1* M2 | *ISA1* M3 | *PUL* M1 | *PUL* M2 | *PUL* M3 | *PUL* M4 | *PUL* M5 | *PUL* M6 |
| --- | --- | --- | --- | --- | --- | --- | --- | --- | --- | --- | --- | --- | --- | --- | --- | --- | --- | --- | --- | --- | --- | --- | --- | --- | --- | --- | --- | --- | --- | --- | --- | --- | --- | --- | --- | --- | --- | --- | --- | --- | --- | --- | --- | --- | --- | --- |
| 26 | a | a | b | a | a | b | a | b | a | a | a | a | b | a | b | a | a | a | a | a | b | b | c | a | b | b | a | b | a | b | a | b | b | b | a | a | b | a | b | b | a | b | a | b | a | a |
| 27 | b | b | b | a | b | a | b | a | a | a | a | a | b | a | b | b | b | b | b | b | b | a | d | b | a | b | b | a | a | a | b | a | b | a | b | b | a | b | a | a | b | a | b | a | b | b |
| 28 | b | b | b | a | b | a | a | a | b | b | a | a | b | a | b | b | b | b | b | b | b | a | a | b | b | a | b | a | a | a | b | a | b | a | b | b | a | b | a | a | b | c | b | a | b | b |
| 29 | a | a | b | a | a | a | a | b | a | a | a | a | b | b | b | a | b | a | a | a | b | b | a | b | b | a | a | b | b | b | a | b | a | b | a | a | b | a | b | b | a | b | a | b | a | a |
| 30 | b | b | b | a | b | a | b | a | a | a | a | a | b | a | b | b | b | b | b | b | b | a | d | b | a | b | b | a | a | a | b | a | b | a | b | b | a | b | a | a | b | a | b | a | b | b |
| 31 | b | b | b | a | b | a | b | a | a | a | a | a | b | a | b | b | b | b | b | b | b | a | d | b | a | b | b | a | a | a | b | a | b | a | b | b | a | b | a | a | b | a | a | a | b | b |
| 32 | b | b | b | a | b | a | b | a | a | a | a | a | b | a | b | b | b | b | b | b | b | a | d | b | a | b | b | a | a | a | b | a | b | a | b | b | a | b | a | a | b | a | a | a | b | b |
| 33 | a | a | b | a | a | a | a | b | a | a | a | a | b | b | b | a | b | a | a | a | b | b | c | a | b | b | a | b | a | b | a | b | b | b | a | a | b | a | b | b | a | b | b | b | a | a |
| 34 | a | a | b | a | a | a | a | b | a | a | a | a | b | b | b | a | b | a | a | a | b | b | c | a | b | b | a | b | a | b | a | b | b | b | a | a | b | a | b | b | a | b | b | b | a | a |
| 35 | a | a | a | b | a | a | a | b | a | a | a | a | b | b | b | a | b | a | a | a | b | b | c | a | b | b | a | b | a | b | a | b | b | b | a | a | b | a | b | b | a | b | b | b | a | a |
| 36 | c | b | b | a | b | a | a | a | a | a | b | a | a | a | b | b | b | b | b | b | b | a | b | a | b | b | b | a | a | a | b | a | b | a | b | b | a | b | a | a | b | a | a | a | b | b |
| 37 | c | b | b | a | b | a | b | a | a | a | b | a | a | a | b | b | b | b | b | b | b | a | b | a | b | b | b | a | a | a | b | a | b | a | b | b | a | b | a | a | b | a | b | a | b | b |
| 38 | c | b | b | a | b | a | a | b | b | b | a | a | b | a | b | b | b | b | b | b | b | a | d | a | a | b | b | a | a | a | b | a | b | a | b | b | a | b | a | a | b | c | a | b | a | a |
| 39 | c | b | b | a | b | a | a | b | b | b | a | a | b | a | b | b | b | b | b | b | b | a | d | a | b | b | a | a | a | a | b | a | b | a | b | b | a | b | a | a | b | c | a | b | a | a |
| 40 | a | a | a | b | a | a | a | b | a | a | a | a | b | b | b | a | b | a | a | a | b | b | a | b | b | a | a | b | a | b | b | a | b | b | a | a | b | a | b | b | a | b | a | b | a | a |
| 41 | a | a | a | b | a | a | a | b | a | a | a | a | b | b | b | a | b | a | a | a | b | b | a | b | b | a | a | b | a | b | b | a | b | b | a | a | b | a | b | b | a | b | a | b | a | a |
| 42 | c | b | b | a | b | a | b | a | a | a | a | a | b | a | b | b | b | b | b | b | b | a | b | a | b | b | b | a | a | a | b | a | b | a | b | b | a | b | a | b | b | c | a | b | a | a |
| 43 | b | b | b | a | b | a | b | a | a | a | a | a | b | a | b | b | b | b | b | b | b | a | b | a | b | b | b | a | a | a | b | a | b | a | b | b | a | b | a | a | b | a | b | a | b | b |
| 44 | a | a | b | a | b | a | b | b | a | a | a | a | b | a | b | b | b | b | b | b | b | a | b | a | b | b | b | b | a | b | b | a | b | a | b | b | a | b | a | a | b | a | b | a | b | b |
| 45 | c | b | b | a | b | a | b | a | a | a | a | a | b | a | b | b | b | b | b | b | b | a | b | a | b | b | b | a | a | a | b | a | b | a | b | b | a | b | a | a | b | a | b | a | b | b |
| 46 | b | b | b | a | b | a | b | a | a | a | a | a | b | a | b | b | b | b | b | b | b | a | b | a | b | b | b | a | a | a | b | a | b | a | b | b | a | b | a | a | b | a | b | a | b | b |
| 47 | b | b | b | a | b | a | a | a | a | a | b | a | a | a | b | b | b | b | b | b | b | a | b | b | b | b | b | b | a | b | b | a | b | a | a | a | b | b | a | a | b | b | b | a | b | b |
| 48 | a | a | b | a | c | a | a | a | a | a | a | a | b | a | b | b | b | b | b | b | b | a | d | a | a | b | b | a | a | a | b | a | b | b | a | b | a | b | a | a | b | a | b | a | b | b |
| 49 | b | b | b | a | c | a | b | b | a | a | a | a | b | a | b | b | b | b | b | b | b | a | d | a | a | b | b | b | a | b | b | a | b | b | a | a | b | b | a | a | b | a | b | a | b | b |
| 50 | b | b | b | a | b | a | a | a | a | a | a | a | b | a | a | b | b | b | b | b | b | a | d | a | a | b | b | a | a | a | b | a | b | a | b | b | a | b | a | a | b | b | b | a | b | b |

Supplementary Table 7**|** Continued

|  | *AGPlar* M1 | *AGPlar* M2 | *AGPiso* M2 | *AGPiso* M3 | *AGPsma* M1 | *AGPsma* M2 | *GBSSII* | *SSI* M1 | *SSI* M2 | *SSI* M3 | *SSIIa* M1 | *SSIIa* M2 | *SSIIa* M3 | *SSIIb* M1 | *SSIIb* M2 | *SSIIb* M3 | *SSIIc* M1 | *SSIIc* M2 | *SSIIc* M3 | *SSIIc* M4 | *SSIIIa* M1 | *SSIIIa* M2 | *SSIIIb* M1 | *SSIIIb* M2 | *SSIIIb* M3 | *SSIIIb* t1 | *SSIVa* M1 | *SSIVb* M1 | *SSIVb* M2 | *SSIVb* M3 | *SBE1* M1 | *SBE1* M2 | *SBE1* M3 | *BEIIa* M1 | *BEIIa* M2 | *BEIIb* M1 | *BEIIb* t1 | *ISA1* M1 | *ISA1* M2 | *ISA1* M3 | *PUL* M1 | *PUL* M2 | *PUL* M3 | *PUL* M4 | *PUL* M5 | *PUL* M6 |
| --- | --- | --- | --- | --- | --- | --- | --- | --- | --- | --- | --- | --- | --- | --- | --- | --- | --- | --- | --- | --- | --- | --- | --- | --- | --- | --- | --- | --- | --- | --- | --- | --- | --- | --- | --- | --- | --- | --- | --- | --- | --- | --- | --- | --- | --- | --- |
| 51 | b | a | b | a | b | a | a | a | a | a | a | a | b | a | b | b | b | b | b | b | b | a | c | a | b | b | b | a | a | a | b | a | b | a | b | b | a | b | a | a | b | a | b | a | b | b |
| 52 | b | b | b | a | c | a | a | b | a | a | a | a | b | a | b | b | b | b | b | b | b | a | d | a | a | b | b | a | a | a | b | a | b | a | b | b | a | b | a | a | b | b | b | a | b | b |
| 53 | b | b | b | a | b | a | a | a | a | a | a | a | b | a | a | b | b | b | b | b | b | a | c | b | b | b | a | b | a | b | b | a | b | b | a | b | a | b | a | a | b | c | a | b | a | a |
| 54 | b | b | b | a | b | a | b | b | b | b | b | a | a | a | b | b | b | b | b | b | b | a | d | a | a | b | a | a | a | a | b | a | b | a | b | b | a | b | a | a | b | b | a | b | a | a |
| 55 | b | b | b | a | b | a | b | b | b | b | b | a | a | a | b | b | b | b | b | b | b | a | d | a | a | b | a | a | a | a | b | a | b | a | b | b | a | b | a | a | b | a | a | b | a | a |
| 56 | c | b | b | a | b | a | a | a | a | a | a | a | b | a | b | b | b | b | b | b | b | a | c | a | b | b | b | b | a | b | b | a | b | a | b | b | a | b | a | a | b | a | b | a | b | b |
| 57 | c | b | b | a | b | a | b | a | b | b | a | a | b | a | b | b | b | b | b | b | b | a | c | a | b | b | b | a | a | a | b | a | b | a | b | b | a | b | a | a | b | c | a | b | a | a |
| 58 | c | b | b | a | b | a | a | a | a | a | a | a | b | a | b | b | b | b | b | b | b | a | c | a | b | b | b | b | a | b | b | a | b | a | b | b | a | b | a | a | b | a | b | a | b | b |
| 59 | b | b | b | a | b | a | b | a | a | a | a | a | b | a | b | b | b | b | b | b | b | a | c | a | b | b | b | a | a | a | b | a | b | a | b | b | a | b | a | a | b | a | b | a | b | b |
| 60 | a | a | a | b | a | a | a | b | a | a | a | a | b | b | b | a | b | a | a | a | b | b | d | a | b | b | a | b | a | b | a | b | b | b | a | a | b | a | b | b | a | b | a | b | a | a |
| 61 | a | a | a | b | a | a | a | a | a | a | a | a | b | b | b | a | b | a | a | a | b | b | d | a | b | b | a | b | a | b | a | b | b | b | a | a | b | a | b | b | a | b | a | b | a | a |
| 62 | a | a | a | b | a | a | a | a | a | a | a | a | b | b | b | a | b | a | a | a | b | b | d | a | b | b | a | b | a | b | a | b | b | b | a | a | b | a | b | b | a | b | a | b | a | a |
| 63 | a | a | b | a | a | a | a | a | a | a | a | a | b | b | b | a | a | a | b | b | b | b | d | a | b | b | a | b | a | b | a | b | b | b | a | a | b | a | b | b | a | b | a | b | a | a |

a, b, c, d indicated the types of genetic polymorphisms in 63 glutinous rice accessions under specific molecular markers.
